# Intense endoplasmic reticulum stress (ERS) / IRE1α enhanced Oxaliplatin efficacy by decreased ABCC10 in colorectal cancer cells

**DOI:** 10.1186/s12885-022-10415-8

**Published:** 2022-12-30

**Authors:** Xiaohui Liu, Bo Wu, Hong Chen, Haimei Sun, Xiaoxia Guo, Tingyi Sun, Deshan Zhou, Shu Yang

**Affiliations:** 1 Department of Histology and Embryology, School of Basic Medical Sciences, Capital Medical University, Beijing, 100069 China; 2 Beijing Key Laboratory of Cancer Invasion and Metastasis Research, Beijing, 100069 China; 3 Experimental Center for Basic Medical Teaching, Capital Medical University, Beijing, 100069 China

**Keywords:** ABCC10, Colorectal cancer, Endoplasmic reticulum stress, IRE1α, Oxaliplatin, Unfolded protein response

## Abstract

**Background:**

Attenuated Oxaliplatin efficacy is a challenge in treating colorectal cancer (CRC) patients, contributory to the failure in chemotherapy and the risks in relapse and metastasis. However, the mechanism of Oxaliplatin de-efficacy during CRC treatment has not been completely elucidated.

**Methods:**

Microarray screening, western blot and qPCR on clinic CRC samples were conducted to select the target gene ABCC10 transporter. The Cancer Genome Atlas data was analyzed to figure out the correlation between the clinical manifestation and ABCC10 expression. ABCC10 knock-down in CRC cells was conducted to identify its role in the Oxaliplatin resistance. Cell counting kit-8 assay was conducted to identify the CRC cell viability and Oxaliplatin IC_50_. Flow cytometry was conducted to detect the cell apoptosis exposed to Oxaliplatin. The intracellular Oxaliplatin accumulation was measured by ultra-high performance liquid chromatography coupled to tandem mass spectrometry.

**Results:**

CRC patients with higher ABCC10 were prone to relapse and metastasis. Differential ABCC10 expression in multiple CRC cell lines revealed a strong positive correlation between ABCC10 expression level and decreased Oxaliplatin response. In ABCC10 knock-down CRC cells the Oxaliplatin sensitivity was evidently elevated due to an increase of intracellular Oxaliplatin accumulation resulted from the diminished drug efflux. To explore a strategy to block ABCC10 in CRC cells, we paid a special interest in the endoplasmic reticulum stress (ERS) / unfolded protein response (UPR) that plays a dual role in tumor development. We found that neither the inhibition of ERS nor the induction of mild ERS had anti-CRC effect. However, the CRC cell viability was profoundly decreased and the pro-apoptotic factor CHOP and apoptosis were increased by the induction of intense ERS. Significantly, the Oxaliplatin sensitivity of CRC cells was enhanced in response to the intense ERS, which was blocked by inhibiting IRE1α branch of UPR. Finally, we figured out that the intense ERS down-regulated ABCC10 expression via regulated IRE1-dependent decay activity.

**Conclusion:**

Oxaliplatin was a substrate of ABCC10 efflux transporter. The intense ERS/IRE1α enhanced Oxaliplatin efficacy through down-regulating ABCC10 in addition to inducing CHOP. We suggested that introduction of intense ERS/UPR could be a promising strategy to restore chemo-sensitivity when used in combination with Oxaliplatin or other chemotherapeutic drugs pumped out by ABCC10.

**Supplementary Information:**

The online version contains supplementary material available at 10.1186/s12885-022-10415-8.

## Background

According to the latest global cancer burden data released by the International Agency for Research on Cancer in 2020, the number of new cases of colorectal cancer (CRC) reached approximately 1.93 million worldwide, and the death toll 935,173 [1]. Some early or local CRC are amendable to appropriate endoscopic or surgical resection only. For advanced or metastatic CRC, systemic therapy typically including a chemotherapy backbone is strongly proposed either going to be curative or palliative [2]. At present, Fluorouracil in combination with Oxaliplatin confers encouraging overall survival advantage in metastatic CRC and, as adjuvant therapy, in some stage II and most stage III patients. However, drug resistance reportedly develops in nearly all advanced CRC patients, resulting in a poor 5-year survival rate in advanced CRC patients, especially for stage IV CRC patients [3].

The development of chemo-resistance is a complex process achieved through either intrinsic or acquired ways, in which acquired over-expression of ATP-binding cassette transporters (ABC transporters) is considered the leading mechanism [4]. The ABC transporter superfamily includes 49 members divided into 7 subfamilies, ABCA ~ ABCG, mediating the efflux of endogenous and exogenous substances [5]. Over-expressed in several chemo-resistant cancer types, ABC transporters confer resistance to various chemotherapeutics through rapid elimination, thereby decreasing their overall accumulation within the cancer cells [6–9]. Twelve ABC transporters were reportedly responsible for drug efflux in humans, among which ABCB1 (P-gp), ABCC1 (MRP) and ABCG2 (BCRP) had a great significance in the efflux of a variety of drugs [10]. Inhibitors of ABC transporters were developed with the demand, which, however, initially showed promise but failed clinically, due to high toxicity and other undesired side effects [11–13]. Hence, developing safe and effective reagents that can block the activity of ABC transporters in cancer cells has a great clinical significance.

The endoplasmic reticulum (ER) is responsible for protein folding, modification and assembly, which are rigorously controlled and highly sensitive to perturbation of ER homeostasis. Under cellular stresses, protein folding is disrupted and leads to an accumulation of unfolded/misfolded proteins, often referred to as ER stress (ERS). To relieve ERS, the unfolded protein response (UPR) is triggered principally through the activation of 3 ERS sensors: inositol requiring enzyme 1α (IRE1α), protein kinase RNA-activated-like ER kinase (PERK) and activating transcription factor 6 (ATF6). The ERS sensors are bound to the glucose-related protein 78 (GRP78) and maintained inactivated under homoeostatic condition. During ERS, GRP78 dissociates from each of the sensors allowing for the activation the UPR. The UPR attempts to re-establish ER homeostasis by reducing incoming protein load, facilitating protein folding and eliminating unfolded proteins. However, if the ERS persists and the UPR is insufficient to deal with the increasing unfolded protein load, the cells would switch from an adaptive response to an apoptotic response, triggering cell death by increasing the pro-apoptotic factor CCAAT/enhancer-binding protein homologous protein (CHOP) [14, 15]. So that it is not surprising that ERS/UPR plays a dual role in deciding the fate of the cancer cells, either facilitating the tumor development or preventing it [16, 17]. Emerging evidence also suggested an implication of ERS/UPR in the chemo-sensitization. Chemo-resistant cancer cells re-gained sensitivity to chemotherapies when either the 3 arms of UPR was knocked down [14]. But paradoxical outcomes were also reported that the activated IRE1α-XBP1s axis and PERK restored sensitivity in myeloma and CRC cells, respectively [18, 19]. Due to the contradictory results, it needs to be further investigated to improve our current knowledge in order to develop ERS/UPR-targeted approaches for treating chemo-resistant CRC.

In this study, we investigated the role of ABCC10 in Oxaliplatin efflux transport in CRC cells and explored the effect of ERS/UPR on ABCC10 expression. The results revealed that the intense ERS was able to restore Oxaliplatin sensitivity in CRC cells through down-regulating ABCC10 via IRE1α pathway.

## Materials and methods

### Clinical samples and microarray

A total of 30 (18 males and 12 females, aged 35 ~ 81 years, without preoperative radiotherapy and chemotherapy) pairs of CRC specimens and adjacent para-tumoral normal tissues were collected from patients who underwent surgical resection at the Beijing Friendship Hospital, Capital Medical University from January, 2005 to December, 2012 (Supplementary information, Table S1). All cases were reviewed by a pathologist and diagnosed as CRC. Microarray analysis was performed on randomly selected 5 pairs of specimens using Affymetrix Clariom D Human with the support from Beijing Cnkingbio Biotechnology Corporation (China) (Supplementary information, Table S1). Briefly, the total RNA was extracted using TRIzol reagent (Life Technologies, USA) and purified with an RNeasy Mini Kit (Qiagen, Germany) according to the manufacturer’s protocol. The RNA quantity and purity were determined by a NanoDrop 2000 spectrophotometer (Thermo Fisher Scientific, USA) at the absorbance of 260 nm. The mRNA expression profiling was measured using Affymetrix Clariom D Human. RNA labeling, microarray hybridization and scanning were performed according to the manufacturer’s instructions. The arrays were scanned using the Gene-Chip® scanner 3000 (Affymetrix, USA). GeneChip operating software 1.4 was then used to analyze the array data. The differentially expressed genes were defined as > twofold change.

### Bioinformatics

Gene expression and clinic data in CRC patients was analyzed based on the Cancer Genome Atlas (TCGA) data from cBioPortal and GEPIA2.

### Cell culture

CRC cell lines including HT-29 (ATCC HTB-38, RRID:CVCL_0320), HCT-116 (ATCC CCL-247, RRID:CVCL_0291), RKO (ATCC CRL-2577, RRID:CVCL_0504), LS174T (ATCC CL-188, RRID:CVCL_1384) and Caco-2 (ATCC HTB-37, RRID:CVCL_0025) were purchased from the American Type Culture Collection (ATCC). Normal colonic epithelial cell line NCM460 (ZKC1143-1, RRID:CVCL_0460) was purchased from the Beijing Zoman Biotechnology Co., Ltd. CRC cells and NCM460 cells were cultured in Dulbecco’s Modi-fied Eagle’s Medium high glucose (DMEM, Gibco, USA) and Roswell Park Memorial Institute (RPMI)-1640 medium (Gibco), respectively, supplemented with 10% of fetal bovine serum (FBS, Biological Industries, Israel), 100 U/mL of peni-cillin–streptomycin (Gibco) and 1% of mycoplasma removal agent Myco-3 (Applichem, German) during experiments. Cells were grown at 37 ℃ in the presence of 5% CO_2_.

### Reagents

4-phenylbutyric acid (4-PBA), a specific ERS antagonist, was purchased from Sigma-Aldrich (P21005, USA). Tunicamycin (Tm), a ERS inducer, was purchased from Cell Signaling Technology (CST, USA, #12,819). STF-083010, an IRE1α-specific inhibitor, was purchased from MedChemExpress (MCE, China, HY-15845). 4-PBA, Tm and STF-083010 were dissolved in dimethylsulfoxide (DMSO, Sigma-Aldrich).

### Cell counting kit-8 assay

Cell viability was assessed using a cell counting kit-8 (CCK-8, Dojindo Laboratories, Japan, CK04) assay according to the manufactory’s instruction. The optical density (OD) values were measured at 450 nm with a microplate reader (Fluoroskan Ascent, Thermo Fisher Scientific). The cell viability was expressed as percentage of the vehicle controls and the IC_50_ values (half maximal inhibitory concentration) were calculated. All experiments were performed in 6 repeats.

### Apoptosis analysis by flow cytometry

CRC cells were stained with Alexa® Fluor 488 Annexin V/PI kit (Invitrogen, USA, V13241) according to the manufacturer’s instruction. Apoptosis was analyzed using a BD FACSCyte flow cytometer (Beckman Coult, USA). All experiments were performed in triplicate.

### Western blot

Total protein was extracted with RIPA lysis (Applygen, China) and subjected to sodium dodecyl sulfate–polyacrylamide gel electrophoresis (SDS/PAGE). PVDF membranes (Millipore, USA) were incubated with appropriate primary antibody overnight at 4 ℃. The primary antibodies included rabbit polyclonal anti-GRP78 (1: 1000, Abcam, USA, ab21685, RRID:AB_2119834), rabbit monoclonal anti-IRE1α (1:1000, CST, #3294, RRID:AB_823545), mouse monoclonal anti-DDIT3/CHOP (1:1000, Abcam, ab11419, RRID:AB_298023) and rabbit polyclonal anti-ABCC10 (1:1000, Abcam, ab107053, RRID:AB_10864747). Mouse monoclonal anti-β-actin (Santa Cruz, USA, sc-8432, RRID:AB_626630) was used as the internal control. The membranes were then incubated with corresponding secondary antibody at room temperature for 2 h. The immunoblotting bands were detected using an enhanced chemiluminescence (ECL) reagent (Thermo Fisher Scientific) on a Fusion FX Vilber Lourmat (France). Image J software (NIH, USA) was employed for densitometric analysis of the immunoblotting bands.

### Quantitative reverse transcription PCR (qRT-PCR)

Total RNA was extracted using TRIzol reagent (Sigma-Aldrich). Reverse transcription of extracted mRNA was performed using a 5 × All-In-One RT MasterMix (ABM, USA). qPCR was performed using a Powerup SYBR Master Mix (Thermo Fisher Scientific) and detected on an ABI7500 qPCR instrument (Applied Biosystems, USA). The reactions were incubated at 95 ℃ for 2 min, followed by 40 cycles of 95 ℃ for 15 s and 60 ℃ for 1 min. All reactions were performed in triplicate. The relative mRNA expression was calculated using the 2^−ΔΔCt^ method. GAPDH was used as the internal control. The oligonucleotide sequences of the qPCR primers are listed in Supplementary information, Table S2.

### siRNA transfection

siRNA specific to ABCC10 (siABCC10) as well as negative control siRNA (siControl) was ordered from RiboBio Co., Ltd. (China). Cells were transfected with siRNA using Opti-MEM I reduced serum medium (Gibco) and Lipofectamine 2000 transfection reagent (Invitrogen) according to the manufacturers’ instructions. Twenty four hours post transfection, the knock-down efficacy of ABCC10 was evaluated by qRT-PCR and Western blot (Supplementary information, Fig. S1). CRC cells were further treated with Oxaliplatin for another 24 h.

### Lentiviral shRNA infection

Lentiviral shRNA to ABCC10 (shABCC10) as well as control shRNA (shControl) was constructed by the support from Sangon Biotech (China). Three days post lentiviral shRNA infection, the infection efficiency was evaluated by observing the GFP fluorescence with a fluorescence microscope (Leica DMI3000B, Leica, German). The knock-down efficacy of ABCC10 was evaluated by qRT-PCR and Western blot (Supplementary information, Fig. S1).

### UPLC-MS/MS

Intracellular drug concentration was detected by ultra-high performance liquid chromatography coupled to tandem mass spectrometry (UPLC-MS/MS). A Waters Acquity UPLC H-Class system (USA) was used for liquid chromatography. The chromatographic separation was performed on a ACQUITY UPLC HSS T3 column (100 mm × 2.1 mm, 1.8 μm) and the temperature was maintained at 30 ℃. The mobile phase consisted of acetonitrile:water (7:93, for Oxaliplatin detection) or methyl alcohol:water (55:45, for Paclitaxel detection) at a flow rate of 0.3 mL/min. The injection volume was 10 μL. MS/MS analysis was performed on a Waters Xevo TQ-XS triple quadrupole mass spectrometer.

### Statistical analysis

Results were presented as the mean ± SEM. For relative quantification, the value of the control group was considered 1 or 100%. Student *t*-test, ANOVA followed by Tukey’s post hoc test and Pearson correlation were conducted using GraphPad prism 8 (GraphPad Software, USA) and SPSS20.0 (IBM, USA). *P*-value of 0.05 or less was considered statistically significant. GraphPad prism 8 and Photoshop CC 2017 (Adobe, USA) were used to create the artwork.

## Results

### ABCC10 was associated with Oxaliplatin response and CRC malignancy

**Fig. 1:**
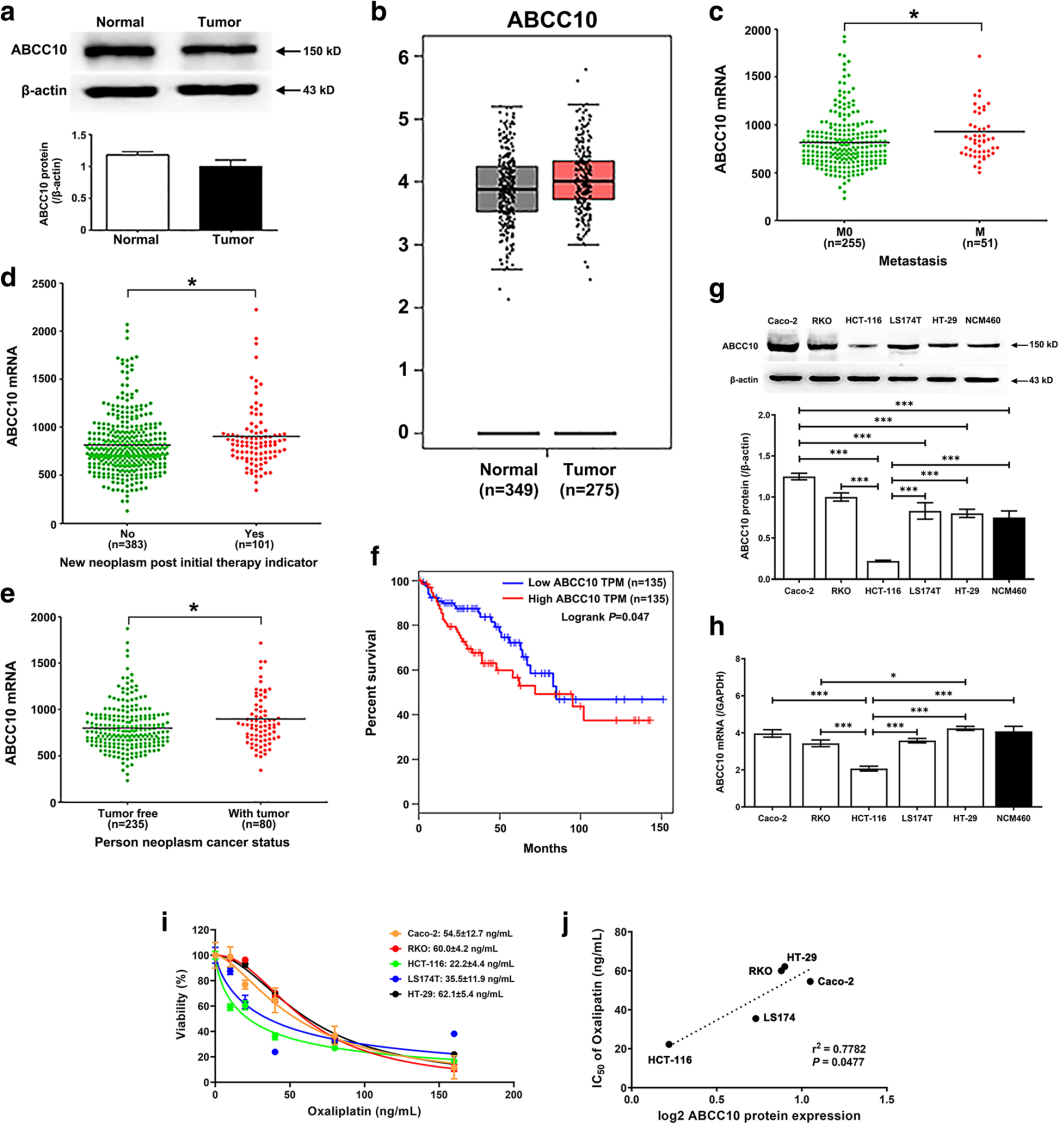
ABCC10 is positively correlated with IC50 of Oxaliplatin and contributes to CRC malignancy. **a** ABCC10 protein is undifferentiated between CRC tissues and adjacent normal mucosal tissues. *n* = 30. Uncropped blots are provided in Supplementary information Fig. S9. **b** There is no obvious difference in ABCC10 mRNA level between CRC tissues and normal mucosa by GEPIA2 data analysis. **c** CRC patients with higher ABCC10 are prone to metastasis. M0: no metastasis, M: metastasis. * *P* < 0.05. **d** CRC patients with higher ABCC10 are prone to recurrence post initial therapy. * *P* < 0.05. **e** ABCC10 level in CRC patients with tumor is significantly higher when compared with those without tumor. * *P* < 0.05. (f) CRC patients with lower ABCC10 survive those with higher ABCC10 by GEPIA2 data analysis. **g** The basic ABCC10 protein expression in 5 CRC cell lines and NCM460 normal colonic epithelial cell line. *n* = 3. *** *P* < 0.001. Uncropped blots are provided in Supplementary information Fig. S10. **h** The basic ABCC10 mRNA expression in 5 CRC cells lines and NCM460 normal colonic epithelial cell line. *n* = 3. * *P* < 0.05, *** *P* < 0.001. **i** The CRC cells are treated with a gradient of Oxaliplatin (10, 20, 40, 80 and 160 ng/mL) for 24 h. The IC50 of Oxaliplatin in 5 CRC cell lines is calculated according to the cellular viability. *n* = 6. **j** Pearson correlation indicates a positive correlation between the ABCC10 protein level and IC50 of Oxaliplatin in 5 CRC cell lines. *n* = 6

The hyposensitivity to chemotherapeutics in cancer cells primarily attributed to the over-expression of the ABC transporter superfamily. Among these ABC transporters, we paid a special interest in ABCC10 because of the following reasons: ① An analysis on 1444 single nucleotide polymorphisms (SNPs) from a cohort of 623 stage II-IV CRC patients pointed out ABCC10 could be predictive to identify the patients who were more likely to benefit from Oxaliplatin treatment [20]. ② Based on the analysis on TCGA and GEPIA2 database and the clinic CRC specimens, ABCC10 stood out from several other ABC transporter members because that despite there was no significant difference in the ABCC10 expression between the tumors and adjacent normal tissues (Fig. 1a and b), the CRC patients with higher ABCC10 were apt to have metastasis, recurrence and shorter survival time (Fig. 1c ~ f; Supplementary information Fig. S2 ~ S4). ③ The cell viability exposed to Oxaliplatin was assessed and the IC_50_ was calculated. The IC_50_ of Oxaliplatin in the 5 CRC cells including Caco-2, RKO, HCT-116, LS174T and HT-29 was 54.5 ± 12.7 ng/mL, 60.0 ± 4.2 ng/mL, 22.2 ± 4.4 ng/mL, 35.5 ± 11.9 ng/mL and 62.1 ± 5.4 ng/mL, respectively, which was positively correlated with the ABCC10 protein level (Fig. 1g ~ j), indicating that the CRC cells with lower ABCC10 were more Oxaliplatin sensitive.

### Silencing of ABCC10 sensitized CRC cells to Oxaliplatin

**Fig. 2:**
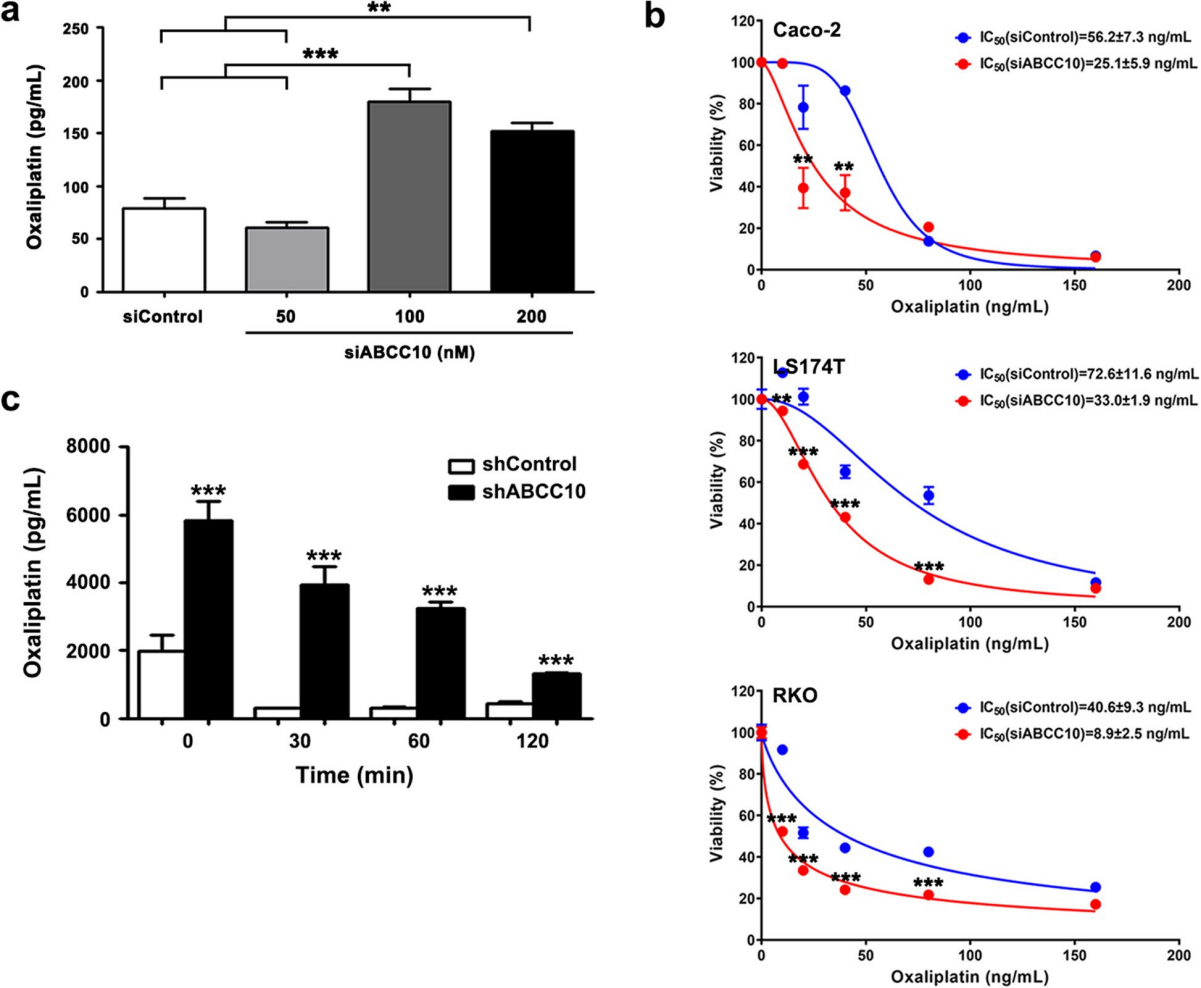
Oxaliplatin is a substrate of ABCC10 and silencing of ABCC10 sensitizes CRC cells to Oxaliplatin. **a** Oxaliplatin accumulation in Caco-2 cells exposed to Oxaliplatin (80 ng/mL) for 24 h is measured by UPLC-MS/MS. The remained intracellular Oxaliplatin is significantly increased when ABCC10 is knocked down by 100 nM and 200 nM of siABCC10. *n* = 3. ** *P* < 0.01, *** *P* < 0.001. **b** CRC cells treated with siABCC10 (100 nM) or siControl (100 nM) are incubated in a gradient of Oxaliplatin (10, 20, 40, 80 and 160 ng/mL) for 24 h. Oxaliplatin IC50 in siABCC10 groups is evidently decreased compared with siControl groups. *n* = 6. ** *P* < 0.01, *** *P* < 0.001. **c** Caco-2 cells are incubated in 80 ng/mL of Oxaliplatin for 24 h. Then Oxaliplatin is withdrawn and the cells are cultured for another 0, 30, 60 or 120 min. In ABCC10 knock-down cells, the intracellular Oxaliplatin residual is much more than that in controls. *n* = 3. *** *P* < 0.001

Cells treated with siABCC10 or siControl were exposed to 80 ng/mL of Oxaliplatin for 24 h and the intracellular accumulation of Oxaliplatin in the same amount of cells (1 × 10^7^) was measured by UPLC-MS/MS. Treatment of 100 nM and 200 nM of siABCC10 significantly increased Oxaliplatin accumulation (179.4 ± 8.8 pg/mL and 152.1 ± 5.1 pg/mL, respectively) compared with siControls (77.0 ± 6.5 pg/mL) and 50 nM of siABCC10 (60.6 ± 3.9 pg/mL) (Fig. 2a). In siABCC10 (100 nM) treated Caco-2, LS174T and RKO cells, Oxaliplatin IC_50_ was declined from 56.2 ± 7.3 ng/mL to 25.1 ± 5.9 ng/mL, 72.6 ± 11.6 ng/mL to 33.0 ± 1.9 ng/mL, and 40.6 ± 9.3 ng/mL to 8.9 ± 2.5 ng/mL, respectively (Fig. 2b).

The increase of intracellular Oxaliplatin could be due to a decrease in the efflux of Oxaliplatin and/or an increase in the uptake of Oxaliplatin. To investigate the exact reason, ABCC10 knock-down Caco-2 cells and controls were incubated with 80 ng/mL of Oxaliplatin for 12 h, followed by further culture in Oxaliplatin-free medium for 0, 30, 60 and 120 min. The remained intracellular Oxaliplatin in shABCC10 group was 5814.3 ± 590.4 pg/mL, 3947.1 ± 508.8 pg/mL, 3240.0 ± 200.0 pg/mL and 1305.0 ± 330.0 pg/mL at 0, 30, 60 and 120 min, respectively, which was significantly increased compared with that in shControl group (1985.7 ± 461.5 pg/mL, 307.5 ± 13.6 pg/mL, 315.0 ± 33.3 pg/mL and 440.0 ± 40.8 pg/mL at 0, 30, 60 and 120 min, respectively) (Fig. 2c), demonstrating that the increased accumulation of intracellular Oxaliplatin by ABCC10 down-regulation was fundamentally because of the reduced efflux of Oxaliplatin.

These results indicated that Oxaliplatin was a substrate of ABCC10 transporter and the highly expressed ABCC10 accelerated Oxaliplatin efflux. Down-regulation of ABCC10 could impede Oxaliplatin efflux, which increased the intracellular Oxaliplatin accumulation and conferred the chemo-sensitivity of CRC cells.

### ERS/UPR was activated in CRC

**Fig. 3:**
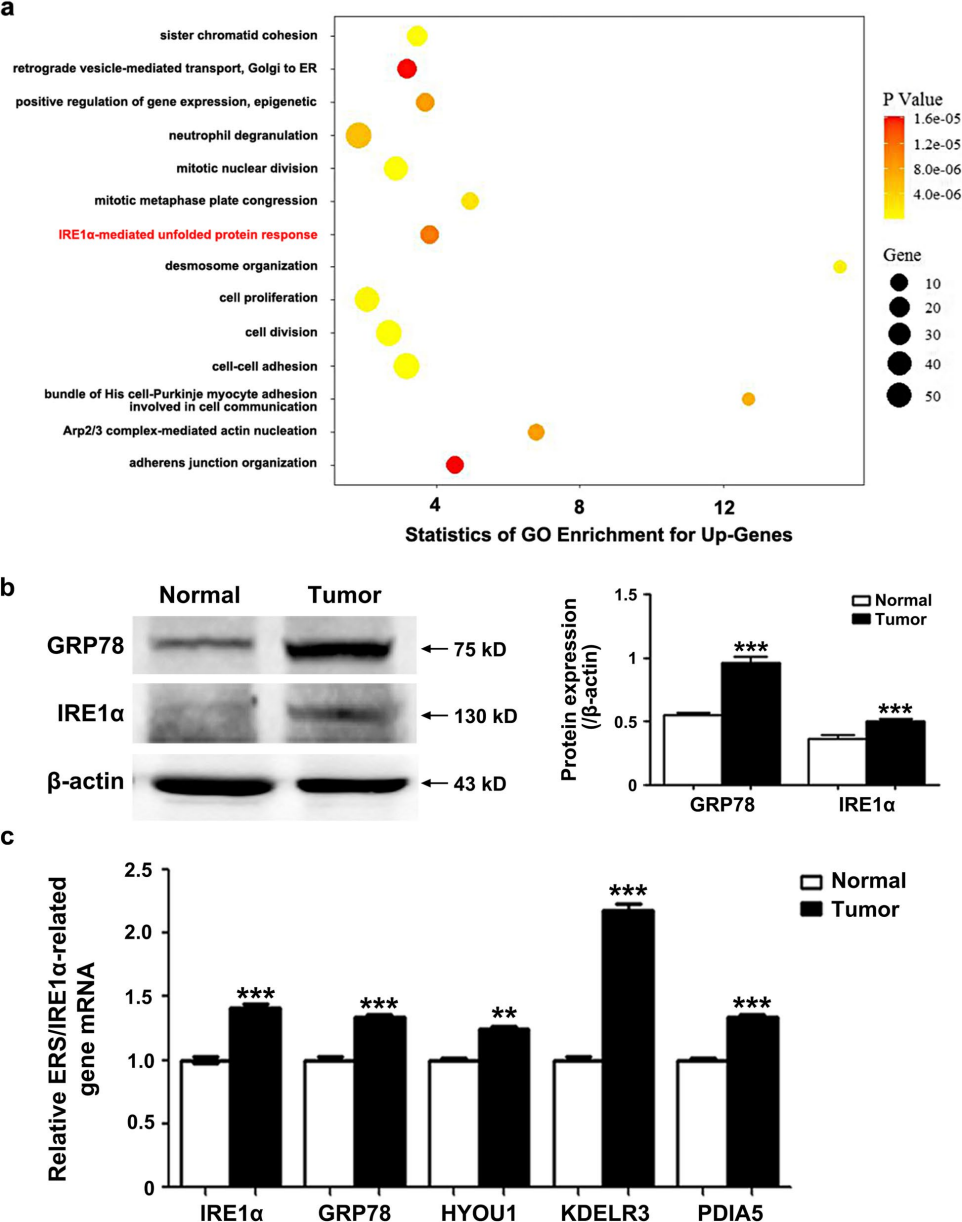
ERS/UPR is activated in CRC tissues. **a** Microarray and GO analysis show that the ERS/UPR, especially the IRE1α pathway, is activated in CRC compared with normal mucosa. **b** The ERS biomarker GRP78 is hyper-expressed and its down-stream IRE1α is activated in CRC. *n* = 6. *** *P* < 0.001. Uncropped blots are provided in Supplementary information Fig. S11. **c** A series of genes involved in the IRE1α pathway are up-regulated in CRC. ** *P* < 0.01, *** *P* < 0.001

The ERS/UPR plays a dual role in tumor development, but its role in the Oxaliplatin resistance has not been fully elucidated. Here, the microarray and GO Enrichment analysis on CRC specimens revealed that the ERS/UPR, especially the IRE1α pathway, was activated in the CRC tissues compared with the adjacent normal tissues (Fig. 3a). GRP78, the biomarker of ERS, and its downstream IRE1α were hyper-expressed in tumors (Fig. 3b). The same results were obtained from the TCGA analysis showing that multiple genes in the IRE1α pathway were highly expressed in the CRC tissues (Fig. 3c).

### Neither inactivation of ERS nor mild ERS was able to significantly reduce CRC cell viability

**Fig. 4:**
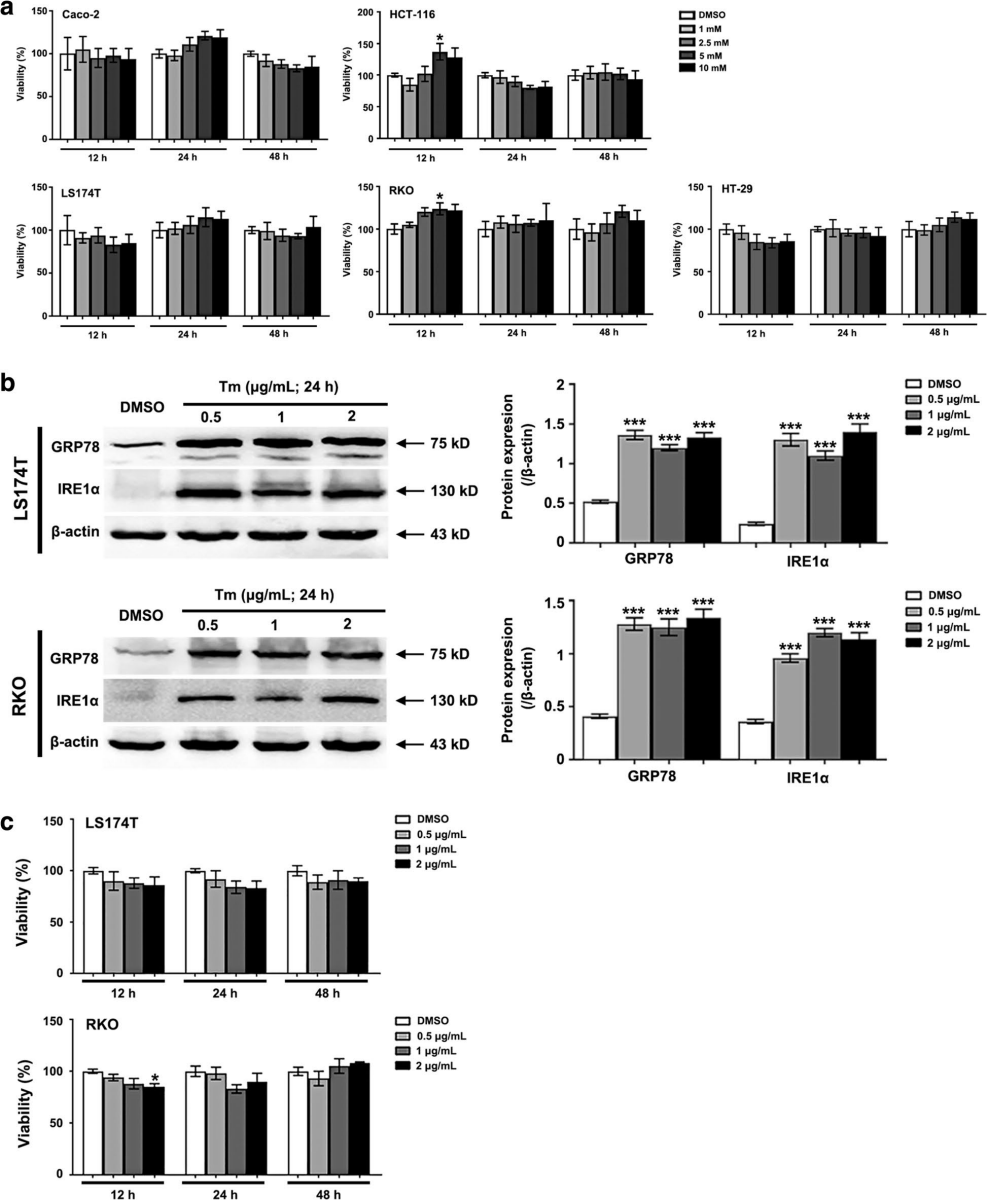
Neither inactivation of ERS nor induction of mild ERS/UPR significantly reduce CRC cell viability. **a** Inhibition of the ERS/UPR by a gradient of 4-PBA (1, 2.5, 5 and 10 mM) for 12 h, 24 h or 48 h dose not suppress the cellular viability significantly. Even, some CRC cells displays increased viability. *n* = 6. * *P* < 0.05. **b** Compared with DMSO, low dose of Tm (0.5, 1 and 2 µg/mL) increases GRP78 and activates IRE1α pathway of the UPR. *n* = 3. *** *P* < 0.001. Uncropped blots are provided in Supplementary information Fig. S12. **c** Except for the treatment of 2 µg/mL of Tm on RKO cells for 12 h, the cellular viability is generally not inhibited a lot when treated with low dose of Tm (0.5, 1 and 2 µg/mL). *n* = 6. * *P* < 0.05. Tm denotes Tunicamycin

In tumor cells, the adaptive ERS/UPR was conducive to the proliferation, invasion, angiogenesis and other malignant biological activities [20], prompting that inactivating ERS/UPR seems to be an anti-tumor approach. Therefore, in this study, the CRC cells were treated with an ERS inhibitor, 4-PBA (1, 2.5, 5 and 10 mM), for 12, 24 or 48 h. Unexpectedly, inactivation of ERS was incompetent to reduce CRC cell viability (Fig. 4a).

Given that the ERS/UPR could also exhibit anti-CRC effect, the CRC cells were exposed to different doses of Tm, which can provoke ERS by blocking the N-glycosylation in the post translational modification. As shown in Fig. 4, despite that low dose of Tm (0.5, 1 and 2 µg/mL) induced the ERS and the activation of IRE1α pathway in the CRC cells (Fig. 4b), the cell viability was not influenced significantly or just slightly reduced (Fig. 4c).

### Intense ERS/UPR elicited significant anti-CRC effect

**Fig. 5:**
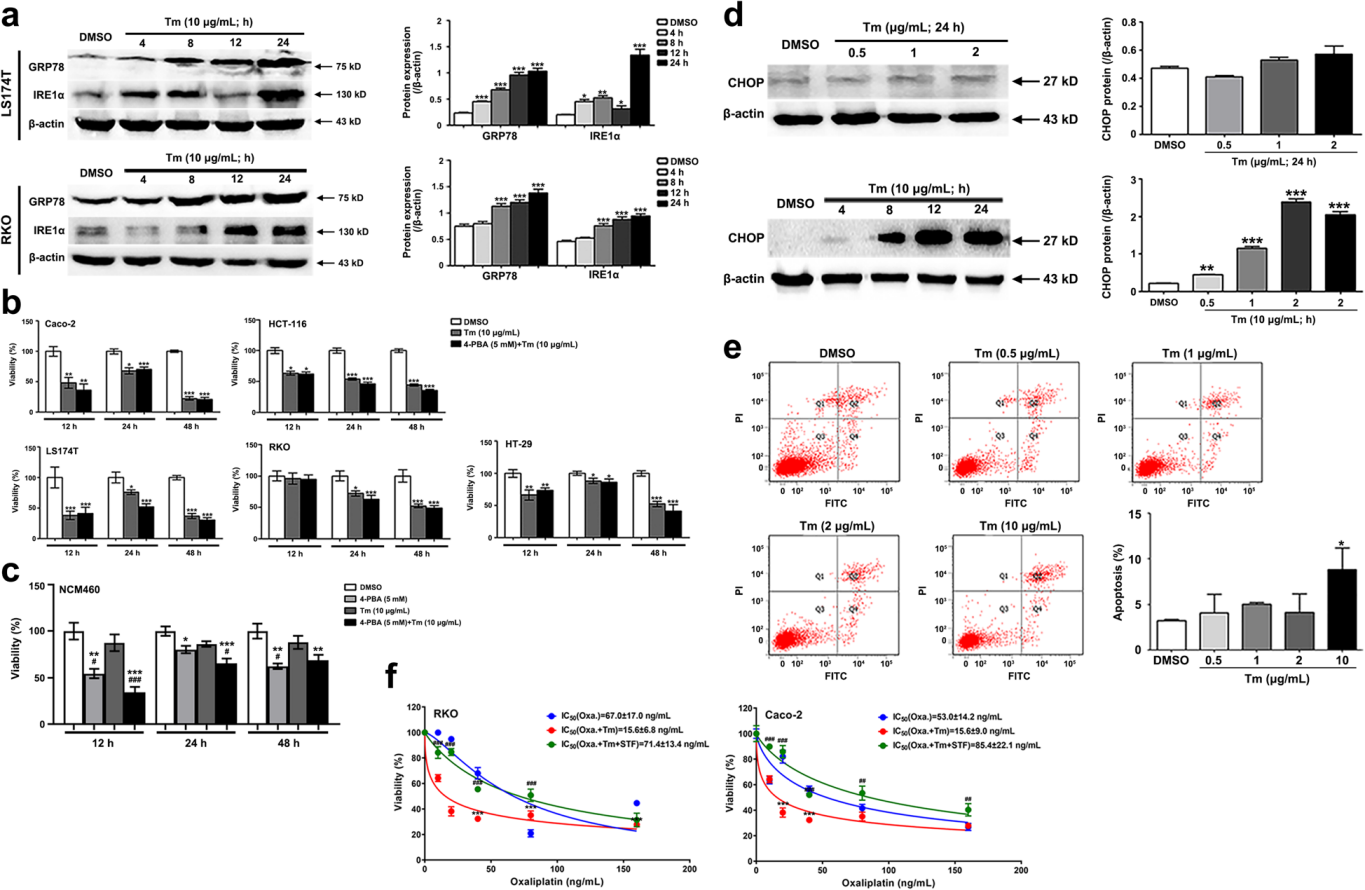
Intense ERS/UPR significantly reduces CRC cell viability, promotes apoptosis and sensitizes CRC cells to Oxaliplatin. **a** High dose of Tm (10 µg/mL) treatment for 4 h, 8 h, 12 h and 24 h increases GRP78 and activates IRE1α pathway. *n* = 3. * *P* < 0.05, ** *P* < 0.01, *** *P* < 0.001. Uncropped blots are provided in Supplementary information Fig. S13. **b** The cellular viability of CRC cells exposed to Tm (10 µg/mL) are remarkably decreased. The additional ERS inhibitor 4-PBA (5 mM) can’t effectively restore the viability. *n* = 6. * *P* < 0.05, ** *P* < 0.01, *** *P* < 0.001. **c** Unlike on the CRC cells, high dose of Tm (10 µg/mL) has no obvious inhibitory effect on the normal human colonic epithelial NCM460 cells; while 4-PBA (5 mM) significantly inhibits their viability. Moreover, the viability of NCM460 cells is kept restrained when co-treated with 4-PBA and Tm. *n* = 6. Compared with DMSO * *P* < 0.05, ** *P* < 0.01, *** *P* < 0.001; Compared with Tm # *P* < 0.05, ### *P* < 0.001. **d** The pro-apoptotic factor CHOP in RKO cells is profoundly increased by high dose of Tm (10 µg/mL) for 4, 8, 12 and 24 h, while kept hypo-expressed upon the treatment of low dose of Tm (0.5, 1 and 2 µg/mL) for 24 h. *n* = 3. ** *P* < 0.01, *** *P* < 0.001. Uncropped blots are provided in Supplementary information Fig. S14. **e** The intense ERS/UPR induced by high dose of Tm (10 µg/mL) significantly accelerates the CRC cell apoptosis; but the mild ERS/UPR induced by low dose of Tm (0.5, 1 and 2 µg/mL) has no such effect. *n* = 3. * *P* < 0.05. **f** The CRC cells are treated with a gradient of Oxaliplatin (10, 20, 40, 80 and 160 ng/mL) for 24 h. Compared with the Oxaliplatin-treated cells, the cell viability is significantly declined in the presence of Tm (10 µg/mL). However, the pre-treatment of STF-083010 (200 µM) for 1 h to block the IRE1α pathway, the declined cell viability is recovered. *n* = 6. Compared with Oxa. *** *P* < 0.001; compared with Oxa. + Tm ## *P* < 0.01, ### *P* < 0.001. Oxa. denotes Oxaliplatin; Tm denotes Tunicamycin, STF denotes STF-083010

On the contrary, high dose of Tm (10 µg/mL) evidently abated the CRC cell viability upon the activation of the ERS/IRE1α pathway (Fig. 5a and b). It is worth noting that the viability of the NCM460 (normal colonic epithelial cell line) was not significantly decreased by high dose of Tm (Fig. 5c). Whereas, inhibition of the ERS by 4-PBA (5 mM) weakened its viability, which was not reversed by the additional Tm (Fig. 5c). Moreover, the intense ERS profoundly up-regulated the pro-apoptotic factor CHOP and promoted the CRC cell apoptosis, while the mild ERS did not (Fig. 5d and e). These results suggested the intense ERS/UPR instead of the mild ERS/UPR could elicit significant anti-CRC effect without obvious side effects on normal colonic epithelial cells.

### Intense ERS/UPR sensitized CRC cells to Oxaliplatin

Next, we wandered if the intense ERS was capable of raising the chemo-sensitivity to Oxaliplatin. The CRC cells were treated with increasing doses of Oxaliplatin in the presence or absence of Tm (10 µg/mL). When treated with Oxaliplatin alone, the IC_50_ was 53.0 ± 14.2 ng/mL in Caco-2 cells and 67.0 ± 17.0 ng/mL in RKO cells (Fig. 5f). The cell viability was further attenuated in response to additional Tm treatment, indicated by the reduced IC_50_ (15.6 ± 9.0 ng/mL in Caco-2 cells and 15.6 ± 6.8 ng/mL in RKO cells) (Fig. 5f). A rescue assay demonstrated that when the IRE1α pathway was blocked by STF-083010 (200 µM), the cell viability was evidently recovered (IC_50_ = 85.4 ± 22.1 ng/mL in Caco-2 cells and IC_50_ = 71.4 ± 13.4 ng/mL in RKO cells) (Fig. 5f). These results suggested that the intense ERS/UPR was competent to enhance Oxaliplatin sensitivity of CRC cells via IRE1α pathway.

### Intense ERS/UPR increased intracellular Oxaliplatin accumulation by down-regulating ABCC10 via IRE1α pathway

**Fig. 6:**
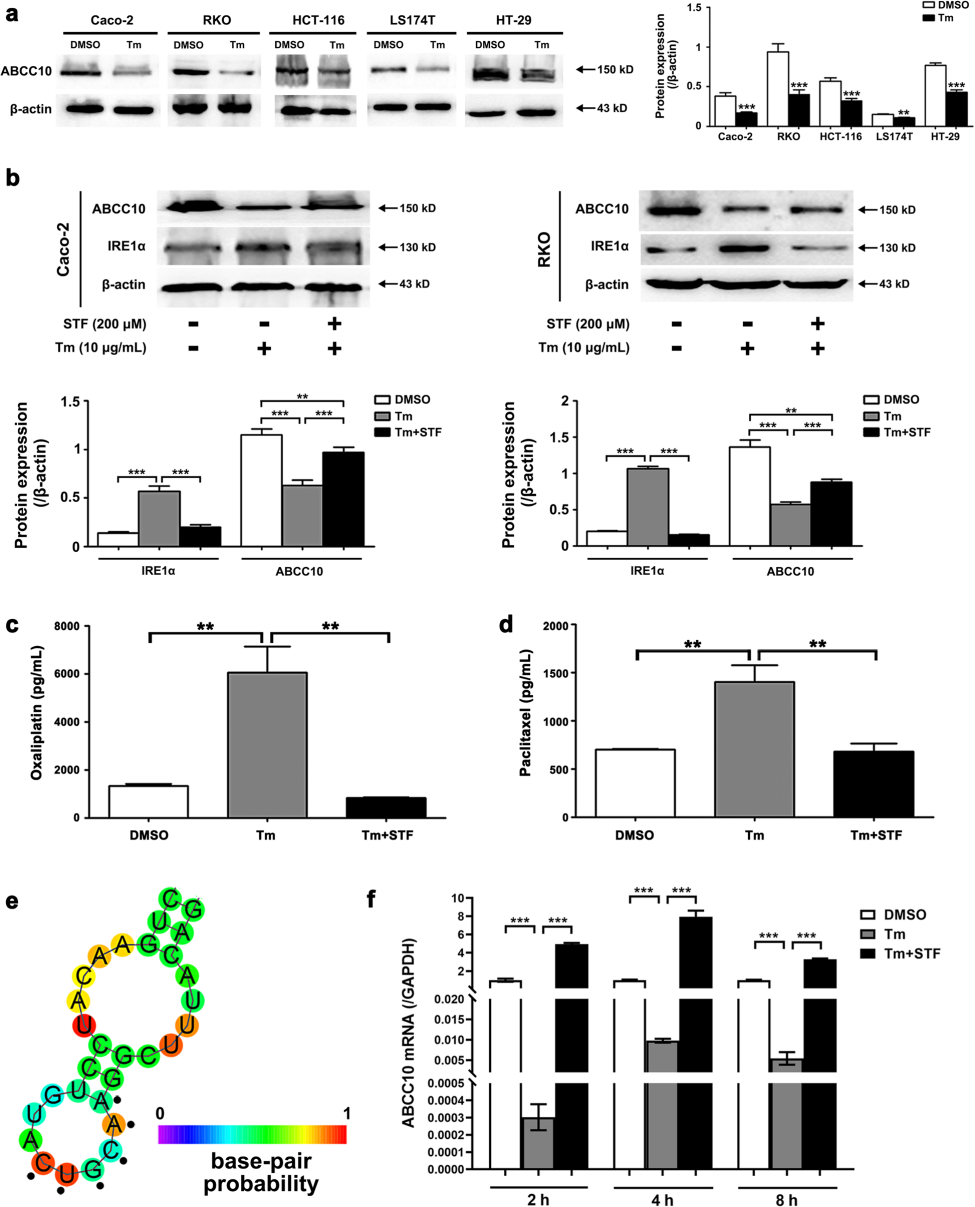
Intense ERS/UPR increases intracellular Oxaliplatin accumulation by down-regulating ABCC10 via IRE1α pathway. **a** High dose of Tm (10 µg/mL) treatment for 24 h clearly decreases ABCC10 in all 5 CRC cell lines. *n* = 3. ** *P* < 0.01, *** *P* < 0.001. Uncropped blots are provided in Supplementary information Fig. S15. **b** High dose of Tm (10 µg/mL) treatment for 24 h activates the IRE1α pathway of UPR and down-regulates ABCC10. While pre-treatment of STF-083010 (200 µM) for 1 h partly recovers ABCC10 expression. *n* = 3. ** *P* < 0.01, *** *P* < 0.001. Uncropped blots are provided in Supplementary information Fig. S16. **c** Oxaliplatin accumulation is significantly increased in response to Tm (10 µg/mL) in Caco-2 cells. While pre-treatment of STF-083010 (200 µM) decreased intracellular Oxaliplatin accumulation. *n* = 3. ** *P* < 0.01. **d** Tm (10 µg/mL) increases intracellular accumulation of Paclitaxel, a determined substrate of ABCC10, which is reversed by pre-treatment of STF-083010 (200 µM). *n* = 3. ** *P* < 0.01. **e** ABCC10 mRNA contains the CUGCAA consensus sequence which forms a hairpin structure predicted by RNAfold Web server. **f** ABCC10 mRNA level is significantly decreased when exposed to Tm (10 µg/mL). Additional STF treatment restores ABCC10 mRNA level up to 8 h. *n* = 3. *** *P* < 0.001. Tm denotes Tunicamycin, STF denotes STF-083010

Since ABCC10 was responsible for Oxaliplatin efflux and sensitivity, we then asked whether ABCC10 was a target of the intense ERS. Analysis on the correlation between ABCC10 mRNA level and XBP-1, a mediator within IRE1α pathway, suggested a negative correlation between them (Supplementary information Fig. S5). As shown in Fig. 6, high dose of Tm (10 µg/mL) significantly down-regulated ABCC10 protein in several CRC cells (Fig. 6a). However, when the IRE1α pathway was blocked by STF-083010 (200 µM), the expression of ABCC10 was restored (Fig. 6b; Supplementary information Fig. S6). The intracellular accumulation of Oxaliplatin in Tm-treated CRC cells reached 6066.7 ± 759.8 pg/mL, which was significantly increased compared with controls (1333.3 ± 47.1 pg/mL). While, the blockage of IRE1α pathway by STF-083010 decreased Oxaliplatin accumulation to 833.3 ± 23.6 pg /mL (Fig. 6c). To confirm the disruptive role of Tm in ABCC10, a determined substrate of ABCC10, Paclitaxel [21], was used as a positive control in this study. Caco-2 cells were exposed to Paclitaxel (80 ng/mL) or Paclitaxel (80 ng/mL) + Tm (10 µg/mL) for 24 h. The intracellular accumulation of Paclitaxel was clearly increased in response to the intense ERS/UPR, but the increase was reversed by pre-treatment of STF-083010 (200 µM) (Fig. 6d).

Finally, we investigated the mechanism by which the intense ERS/UPR decreased ABCC10. Owing to the endoribonuclease domain, IRE1α mediates the cleavage of multiple RNAs in a process known as regulated IRE1-dependent decay (RIDD). The target mRNAs of RIDD contain a consensus sequence, CUGCAA, which could form a hairpin secondary structure in order to be cleaved. By the use of the RNAfold Web server, we found that ABCC10 mRNA (NM_001198934.2) contains the CUGCAA consensus sequence at the site of 1445 ~ 1450, suggesting that ABCC10 mRNA was a putative target of RIDD (Fig. 6e). Thus, we traced ABCC10 mRNA levels in Caco-2 cells exposed to Tm (10 µg/mL) at 2, 4 and 8 h, in the presence or the absence of the IRE1α RNase activity inhibitor STF-083010 (200 µM). ABCC10 mRNA was remarkably decreased when treated with Tm, while STF-083010 increased it up to 8 h (Fig. 6f).

## Discussion

In this study, we for the first time provided evidence that Oxaliplatin is the substrate of ABCC10 transporter. Highly expressed ABCC10 in CRC cells conferred resistance to Oxaliplatin through accelerating drug efflux, contributory to the metastasis and recurrence of CRC. The intense ERS/UPR is capable of down-regulating ABCC10 through IRE1α pathway, which sensitized CRC cells to Oxaliplatin by increasing intracellular drug accumulation.

Oxaliplatin has been commonly used in combination with other chemotherapeutics, especially for those failed in 5-Fluorouracil remedy [22]. However, like most chemotherapeutics, Oxaliplatin resistance usually emerged in surprisingly 90% of metastatic patients. In sensitivity to drugs could be attributed to the decreased drug uptake, impaired DNA adduct formation, alteration in DNA repair genes, defects in cell apoptosis and, the most frequently, over-expression of ABC transporters in tumor cells [23].

CRC cells permanently exposed to a high dose of Oxaliplatin had an up-regulation of ABCB1 and a poor response to Oxaliplatin [24]. Silencing ABCC2 increased Oxaliplatin accumulation and cytotoxicity in Caco-2 CRC cell line [25]. Evidence provided by Ma et al*.* supported the hypothesis that the Oxaliplatin resistance in CRC correlated with the ABCG2 over-expression in a subset of cancer stem cells [26]. In this study, we picked out ABCC10, also known as multidrug resistance-associated protein 7 (MRP7), as a novel putative candidate responsible for Oxaliplatin efflux. In vitro experiments uncovered that ABCC10 conferred tumor cells with multidrug resistance [27–31]. While ABCC10 knock-out mice exhibited susceptibility to Paclitaxel treatment [32]. However, there has been no research demonstrating the role of ABCC10 in Oxaliplatin efflux in CRC cells. Only one analysis based on an investigation of 1444 SNPs from a cohort of 623 stage II-IV CRC patients pointed out that 6 transporter genes, ABCC10 included, could be predictive to identify the patients who were more likely to benefit from Oxaliplatin treatment [20]. Here, we explored that the CRC cells with higher ABCC10 expression were of lower response to Oxaliplatin. With the ABCC10 knock down, the intracellular accumulation of Oxaliplatin was increased and the Oxaliplatin sensitivity was significantly restored consequently.

To date, 3 generations of ABC transporter modulators have been developed to restore chemo-sensitivity or eliminate chemo-resistance. They could either block or inactivate ABC transporters to increase the intracellular concentration of chemotherapeutic drugs within tumor cells. These ABC transporter modulators indeed showed efficacy in pre-clinical trials but always not effective in clinic trials. Some of them produced cardiotoxicity and inhibited hepatic and intestinal cytochrome P450 enzymes thereby causing systemic toxicity [33]. Moreover, nearly all of the ABC transporter modulators are targeting ABCG2, ABCB1, ABCC1 and ABCC2, while ABCC10 modulator has been unavailable yet.

Throughout lifetime, cells are repeatedly faced with a variety of ER stressors, such as inhibition of protein glycosylation, disturbance of calcium ion balance and changes in oxidative stress, that can potentially disrupt ER proteostasis and contribute to the accumulation of cell damage and disease susceptibility. In light of the role of ERS/UPR in the tumor development, we wandered the exact role of ERS/UPR in regulating Oxaliplatin resistance. We found that the intense ERS/UPR instead of the mild ERS/UPR inhibited CRC cell viability and promoted apoptosis with little or no side effect on normal colonic epithelial cells. Actually, some anti-cancer drugs and natural compounds worked through, in part, inducing ERS/UPR. For instance, Nelfinavir, an HIV protease inhibitor, inhibited small-cell lung cancer cell proliferation and induced cell death in vitro and in vivo, which was caused by induction of the UPR [34]. Vitamin E succinate-induced apoptosis was coupled to the ERS/UPR in human gastric cancer cells [35]. Besides drugs, a radiation-induced ERS/UPR has been observed in vivo and in vitro leading to cell dysfunction and subsequent cell death [36]. Furthermore, androstano-arylpyrimidines induced ERS precipitating to decreased multidrug resistance 1 (MDR1)-mediated doxorubicin efflux [37], and lupeol decreased the expression of ABCG2 and activated ERS to induce Oxaliplatin-resistant CRC cell death [38]. These studies indicated that the ERS might be the fundamental mechanism behind the molecular events to defeat drug resistance. Here, our present results shed light on a novel anti-CRC mechanism of intense ERS/UPR by augmenting Oxaliplatin sensitivity and provided evidence for treating CRC patients with Oxaliplatin in combination with ER inducers or radiotherapy. However, the tumor-promoting effect of ERS/IRE1α pathway must not been neglected. IRE1α pathway caused clearance of misfolded proteins in cancer cells and facilitated survival and metastasis. These contradictory roles of IRE1α pathway should be exploited in context of personalized treatment for CRC. We suggest that inducing intense ERS/IRE1α activity could be used for CRC patients with highly expressed ABCC10.

We next investigated how the ERS/UPR multiplied the anti-CRC effect of Oxaliplatin, focusing on its efflux pump, ABCC10. Protein homeostasis, referred to as proteostasis, is under a control of an exquisite network of mechanism in cells. Among them, the ER exerts a substantial effect for it’s the organelle where the protein synthesis, folding, trafficking and degradation are carefully orchestrated. Disrupted protein homeostasis resulted from permanent or intense ERS destroys protein integrity and functionality. But in another way, tumor cell bioactivities could be inhibited as a result of the intense ERS/UPR-induced protein homeostasis perturbation. The ERS decreases protein products from 3 arms of UPR, PERK, ATF6 and IRE1α. IRE1α is a transmembrane protein containing a kinase and an endoribonuclease domain, the latter mediates the RIDD. Degradation of the specific RIDD targeted RNAs may impact particular signaling pathways in a cell-specific manner [39]. In this study, we revealed a RIDD-dependent regulatory mechanism of ABCC10 proteostasis in CRC cells. Previous evidence showed other mechanisms that regulated ABCC10 expression. Transcriptional activation of ABCC10 gene was Cisplatin-induced and histone acetylases EP300-dependent in the presence of p53 [40]. As one of the most common epigenetic RNA modifications, N^6^-methyladenosine (m^6^A) modification mediated the expression of ABCC10. The m^6^A modification was significantly enriched upon ABCC10 gene expression in drug-resistant non-small cell lung cancer [41]. All these results suggested that ABCC10 expression could be mediated via genetic and epigenetic ways. In addition, a number of other ABC transporters responsible for chemo-drug efflux were as well significantly down-regulated by the intense ERS/UPR (Supplementary information, Fig. S7), which means that ABCC10 is not the exclusive target of the ERS. For example, 5-Fluorouracil (5-FU), a conventional anti-CRC drug, is transported by ABCB1 and ABCC5 [42, 43]. As shown in Supplementary information Fig. S8, inducing ERS by Tm could strengthen the inhibitory effect of 5-FU. Besides through down-regulating ABC transporters, ERS does elicit anti-cancer effects through other ways, among which, increasing CHOP, inducing reactive oxygen species (ROS) and provoking JNK pathway are involved [44–46].

Although adjunctive chemotherapy is essential for CRC patients, it is still considered imperfect due to inevitable side effects, such as myelosuppression, ovarian damage, cardiotoxin, hepatotoxicity, nephrotoxicity etc. [16]. Minimizing the side-effects of chemotherapy has been under investigation. An exciting result in this study is that the normal colonic epithelial NCM460 cells keep viable against intense ERS (Fig. 5c), suggesting that the ERS could elicit significant anti-CRC effect without obvious side effects on healthy cells. Based on this preliminary result, other normal cells of the human body need to be examined when exposed to ERS in vitro and in vivo.

## Conclusion

**Fig. 7:**
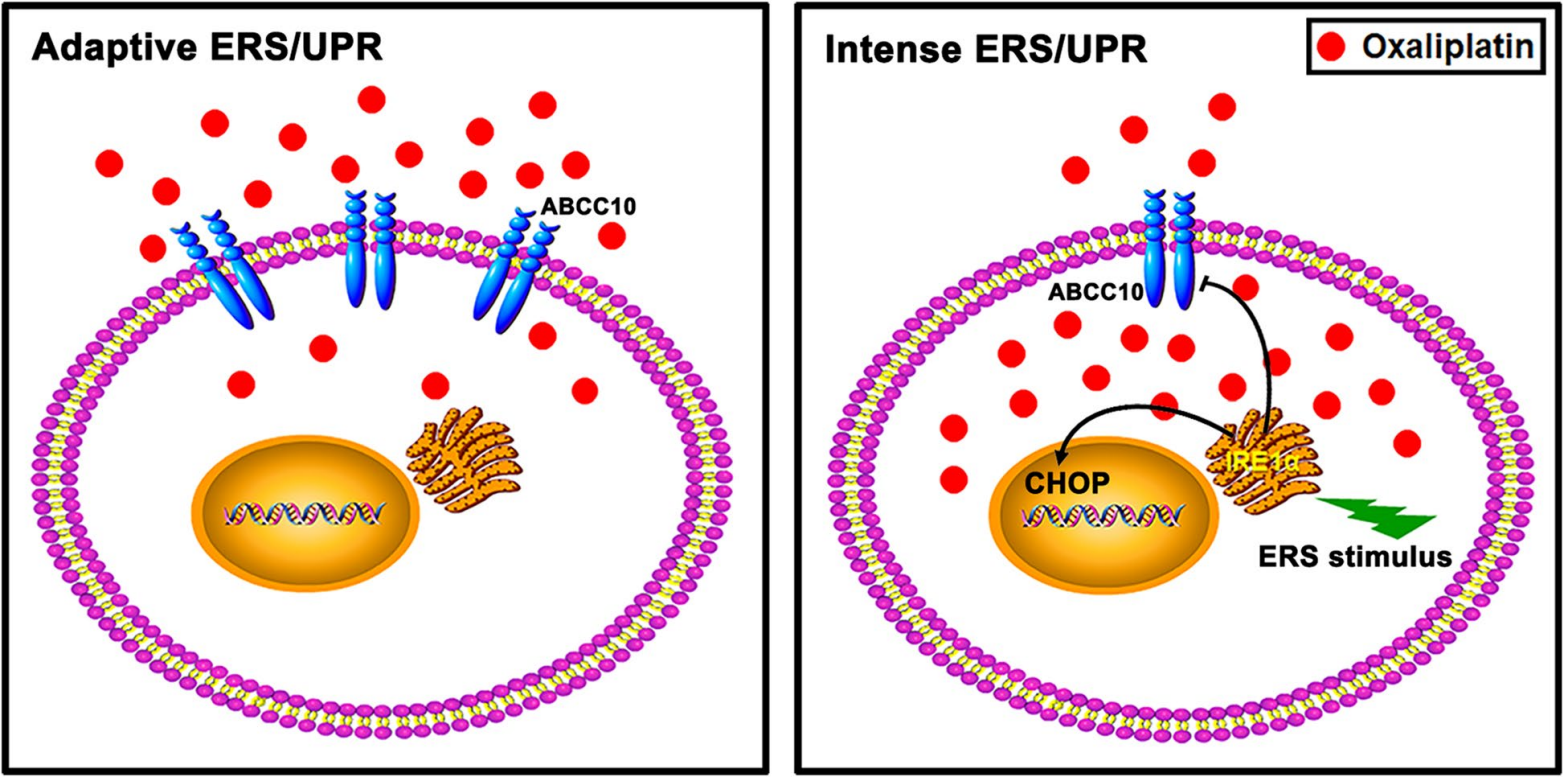
Scheme of intense ERS effect on ABCC10-medicated Oxaliplatin efflux. Oxaliplatin is the substrate of ABCC10, which accelerates efflux of Oxaliplatin so as to reduce its intracellular accumulation. Intense ERS/UPR down-regulates ABCC10 via IRE1α pathway, which increases Oxaliplatin accumulation in CRC cells and consequently sensitizes CRC cells. In addition, intense ERS/UPR up-regulates pro-apoptotic factor CHOP and promotes CRC cell apoptosis. Bolt of lightning denotes ERS stimulus

Our results demonstrated that the intense ERS displayed potent anti-CRC effect partly by sensitizing CRC cells to Oxaliplatin via down-regulating ABCC10 through IRE1α RIDD activity, in addition to increasing pro-apoptotic factor CHOP (Fig. 7). Oxaliplatin in combination with ERS inducer would be better beneficial for CRC patients, especially those refractory to Oxaliplatin.

## Supplementary Information

**Additional file 1:** **Table S1.** Clinic information of CRC patients.

**Additional file 2:** **Table S2.** Oligonucleotide sequences of qPCR primers.

Additional file 3: **Fig. S1.** (a) Three siABCC10 were provided by the manufactory. Twenty four hours post transfection, Western blot and qPCR were conducted to evaluate the ABCC silencing efficacy. ABCC10 protein and mRNA expression is clearly decreased when treated with siABCC10-2 and siABCC10-3. In the further experiments, siABCC10-2 was used. *n*=3. * P.

**Additional file 4: Fig. S2.** Except for the significant higher ABCC3 mRNA and lower ABCC5 mRNA level in the CRC tissues compared with the normal mucosa, none of the mRNA expressions of ABCB1, ABCB9, ABCC1, ABCC2, ABCF2 and ABCG2 is significantly different between CRC tissues and normal mucosa based on the TCGA data analysis. * P.

**Additional file 5: Fig. S3.** (a) None of the mRNA expressions of ABCB1, ABCB9, ABCC1, ABCC2, ABCC3, ABCC5, ABCF2 and ABCG2 is significantly different between the metastasized and non-metastasized CRC patients. M0: no metastasis, M: metastasis. (b) None of the mRNA expressions of ABCB1, ABCB9, ABCC1, ABCC2, ABCC3, ABCC5, ABCF2 and ABCG2 is significantly different between the recurrent and non recurrent CRC patients post initial therapy. (c) None of the mRNA expressions of ABCB1, ABCB9, ABCC1, ABCC2, ABCC3, ABCC5, ABCF2 and ABCG2 in the CRC patients with tumor is significantly higher than those without tumor.

**Additional file 6: Fig. S4.** The percent survival of the CRC patients with higherABCB1, ABCB9, ABCC1, ABCC2, ABCC3, ABCC5, ABCF2 or ABCG2 mRNA is basically the same as those with lower mRNA expression.

**Additional file 7: Fig. S5.** Analysis on the correlation between ABC transporter mRNA level and XBP-1, a mediator within IRE1α pathway, was made based on TCGA data. ABCC10, ABCC5 and ABCF2 negatively correlated with XBP-1.

**Additional file 8: Fig. S6.** Caco-2 and RKO cells were treated with Tm (10 μg/mL) or STF (25, 50, 100, 200 and 400 μM). IRE1α pathway is inactivated upon the treatment of 200 or 400 μM of STF, by which ABCC10 is elevated. Therefore, 200 μM of STF was used in the further experiments.

**Additional file 9: Fig. S7.** Intense ERS/UPR significantly down-regulates mRNA expression of multiple ABC transporters in RKO and HCT-116 cells. RKO and HCT-116 cells are exposed to high dose of Tm (10 µg/mL). ABC transporters including ABCB1, ABCB9, ABCC1, ABCC2, ABCC3, ABCC5, ABCC10, ABCF2 and ABCG2 are remarkably downregulated in Tm-treated HCT-116 cells compared with controls. ABCB1, ABCB9, ABCC1, ABCC2, ABCC3, ABCC5, ABCC10 and ABCG2 were down-regulated in Tm-treated RKO cells while ABCF2 were up-regulated compared with controls. *n*=3. * *P*<0.001.

**Additional file 10: Fig. S8.** RKO and Caco-2 cells were exposed to a gradient of 5-Fluorouracil (5-FU) in the absence or presence of Tm (10 µg/mL) for 24 h. 5-FU IC50 is significantly decreased in response to Tm. *n*=6. * P

**Additional file 11: Fig. S9.** Original data: Uncropped western blot images of Fig. 1a.

**Additional file 12: Fig. S10.** Original data: Uncropped western blot images of Fig. 1g.

**Additional file 13: Fig. S11.** Original data: Uncropped western blot images of Fig. 3b.

**Additional file 14: Fig. S12.** Original data: Uncropped western blot images of Fig. 4b

**Additional file 15: Fig. S13.** Original data: Uncropped western blot images of Fig. 5a.

**Additional file 16: Fig. S14.** Original data: Uncropped western blot images of Fig. 5d.

**Additional file 17: Fig. S15.** Original data: Uncropped western blot images of Fig. 6a.

**Additional file 18: Fig. S16.** Original data: Uncropped western blot images of Fig. 6b. The framed regions of the blots in Fig. S9~S16 are used in the manuscript. Since the blots were cut prior to hybridisation with antibodies, the provided uncropped images are not of full length.

## Data Availability

The datasets generated and/or analyzed during the current study are available in the cBioPortal repository, http://www.cbioportal.org/study/summary?id=coadread_tcga_pan_can_atlas_2018, and the GEPIA2 repository, http://gepia.cancer-pku.cn/detail.php?gene=ABCC10. The microarray data and GO analysis are available from the corresponding author on reasonable request.

## Abbreviations

4-PBA: 4-Phenylbutyric acid
5-FU: 5-Fluorouracil
ABC transporter: ATP-binding cassette transporter
ATF6: Activate transcription factor 6
CCK-8: Cell counting kit-8
CHOP: CCAAT/enhancer-binding protein homologous protein
CRC: Colorectal cancer
ERS: Endoplasmic reticulum stress
GRP78: 78-KDa glucose-regulated protein
IC50: Half maximal inhibitory concentration
IRE1: Inositol-requiring enzyme 1
MRP: Multidrug resistance-associated protein
OD: Optical density
PERK: Protein kinase RNA-activated-like ER kinase
RIDD: Regulated IRE1-dependent decay
Tm: Tunicamycin
UPLC-MS/MS: Ultra-high performance liquid chromatography coupled to tandem mass spectrometry
UPR: Unfolded protein response
XBP1: X-box binding protein 1
