# Supplementary material for: Intense endoplasmic reticulum stress (ERS) / IRE1α enhanced Oxaliplatin efficacy by decreased ABCC10 in colorectal cancer cells
Source: BMC Cancer. 2022 Dec 30;22:1369. doi: 10.1186/s12885-022-10415-8 (PMC9805014; doi:10.1186/s12885-022-10415-8)
Supplement: Supplementary file 1 — Additional file 1: Table S1. Clinic information of CRC patients. [file 12885_2022_10415_MOESM1_ESM.pdf]

Table S1 Clinic information of CRC patients

Patient ID	Age	Sex	Stage	Metastasis	Recurrence
03367247*	35	Male	II	—	No
01266953	40	Female	IV	Ovary	Yes
03317077	44	Male	III	—	Yes
00905381*	44	Female	II	—	No
03496055*	45	Male	III	—	No
03558284	49	Male	III	—	No
2105959	49	Male	III	—	No
03116629	51	Female	II	—	No
3495339	52	Male	II	—	No
1705622	53	Male	III	—	Yes
01764703	56	Female	I	—	No
03350867	57	Female	II	—	No
03227707	57	Male	III	—	Yes
03386847	58	Male	II	—	No
03282975	59	Female	II	—	No
03321116*	64	Male	III	—	No
03496056	67	Male	II	—	No
03472220	68	Male	III	—	No
03553424	72	Female	IV	Lung	No
01686410	76	Female	III	—	No
03090276	61	Male	II	—	No
02360007*	67	Female	II	—	No
00841282	74	Female	II	—	No
02089484	75	Female	I	—	No
00184827	75	Female	III	—	No
01704165	76	Male	III	—	Yes
02091652	77	Male	III	—	Yes
00147940	79	Male	III	—	Yes
00455798	80	Male	II	—	Yes
00469644	81	Male	III	—	Yes

* Randomly selected for microarray analysis.
