# Supplementary material for: Intense endoplasmic reticulum stress (ERS) / IRE1α enhanced Oxaliplatin efficacy by decreased ABCC10 in colorectal cancer cells
Source: BMC Cancer. 2022 Dec 30;22:1369. doi: 10.1186/s12885-022-10415-8 (PMC9805014; doi:10.1186/s12885-022-10415-8)
Supplement: Supplementary file 2 — Additional file 2: Table S2. Oligonucleotide sequences of qPCR primers. [file 12885_2022_10415_MOESM2_ESM.pdf]

Table S2 Primer sequences

| Gene | Sequences |
| --- | --- |
| ABCB1 | Forward: 5′- TTGCTGCTTACATTCAGGTTTCA -3′ |
| | Reverse: 5′- AGCCTATCTCCTGTCGCATTA -3′ |
| ABCB9 | Forward: 5′-ATTGATGGCATCGTCATCCAG-3′ |
| | Reverse: 5′-CGAAGGCGAATGTTCAGTCTG-3′ |
| ABCC1 | Forward: 5′-CTCTATCTCTCCCGACATGACC-3′ |
| | Reverse: 5′-AGCAGACGATCCACAGCAAAA-3′ |
| ABCC2 | Forward: 5′- CCCTGCTGTTCGATATACCAATC -3′ |
| | Reverse: 5′- TCGAGAGAATCCAGAATAGGGAC -3′ |
| ABCC3 | Forward: 5′-TGGGGTGAAGTTTCGTACTGG-3′ |
| | Reverse: 5′-CACGTTTGACTGAGTTGGTGATA-3′ |
| ABCC5 | Forward: 5′-CTCTATCTCTCCCGACATGACC-3′ |
| | Reverse: 5′-AGCAGACGATCCACAGCAAAA-3′ |
| ABCC10 | Forward: 5′- GTCCAGATTACATCCTACCCTGC -3′ |
| | Reverse: 5′- GCCAACACCTCTAGCCCTATG -3′ |
| ABCF2 | Forward: 5′-CCTTCACTTACCTTTTCATGGTC-3′ |
| | Reverse: 5′-ACTTCACGCTTCCCAATAGCA-3′ |
| ABCG2 | Forward: 5′-ACGAACGGATTAACAGGGTCA-3′ |
| | Reverse: 5′-CTCCAGACACACCACGGAT-3′ |
| GAPDH | Forward: 5′- CCTGCACCACCAACTGCTTA -3′ |
| | Reverse: 5′- AGTGATGGCATGGACTGTGG -3′ |
