# Supplementary figures and images for: Intense endoplasmic reticulum stress (ERS) / IRE1α enhanced Oxaliplatin efficacy by decreased ABCC10 in colorectal cancer cells
Source: BMC Cancer. 2022 Dec 30;22:1369. doi: 10.1186/s12885-022-10415-8 (PMC9805014; doi:10.1186/s12885-022-10415-8)

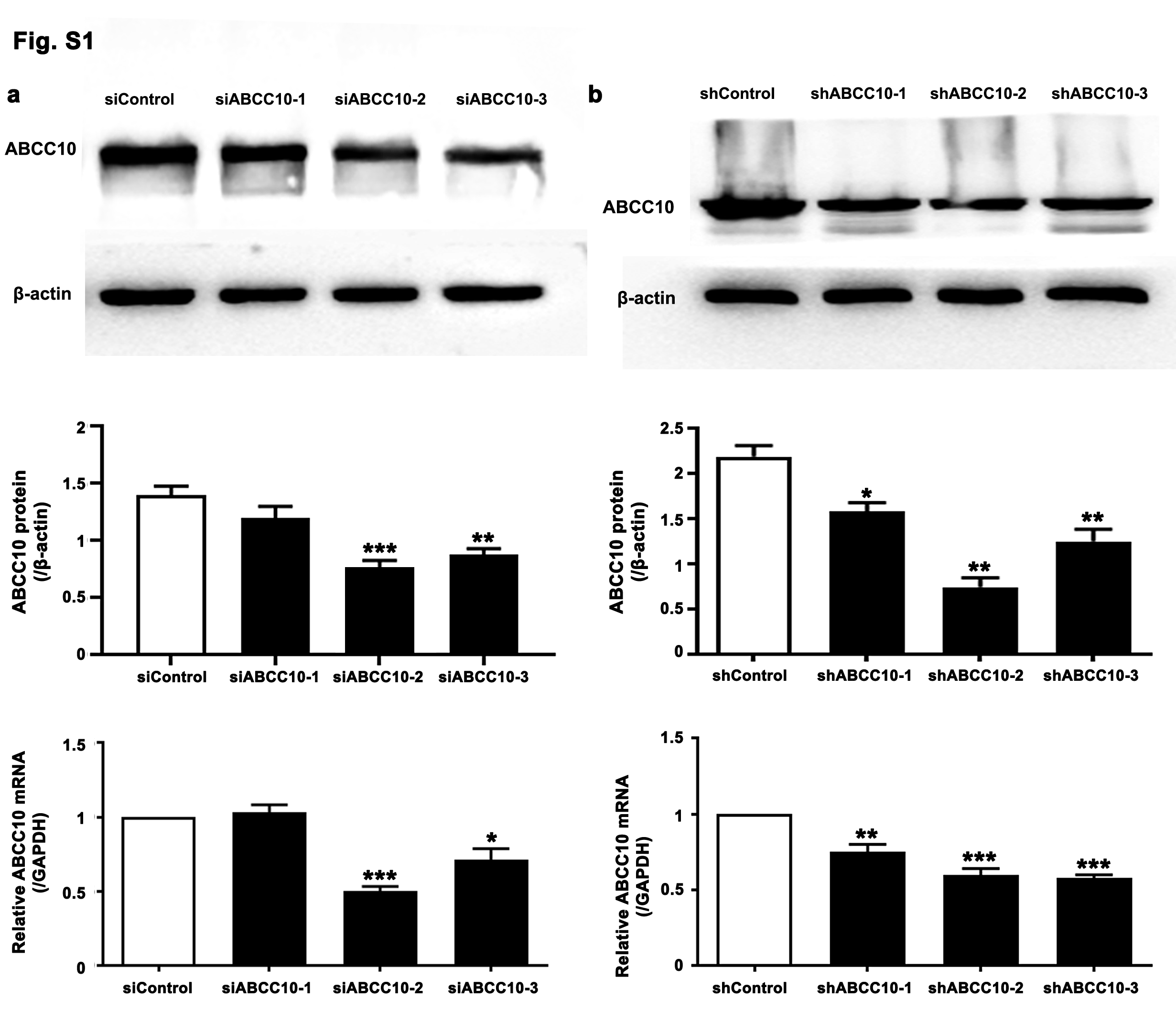

Supplement: Supplementary file 3 — Additional file 3: Fig. S1. (a) Three siABCC10 were provided by the manufactory. Twenty four hours post transfection, Western blot and qPCR were conducted to evaluate the ABCC silencing efficacy. ABCC10 protein and mRNA expression is clearly decreased when treated with siABCC10-2 and siABCC10-3. In the further experiments, siABCC10-2 was used. n=3. * P. [file 12885_2022_10415_MOESM3_ESM.tif]

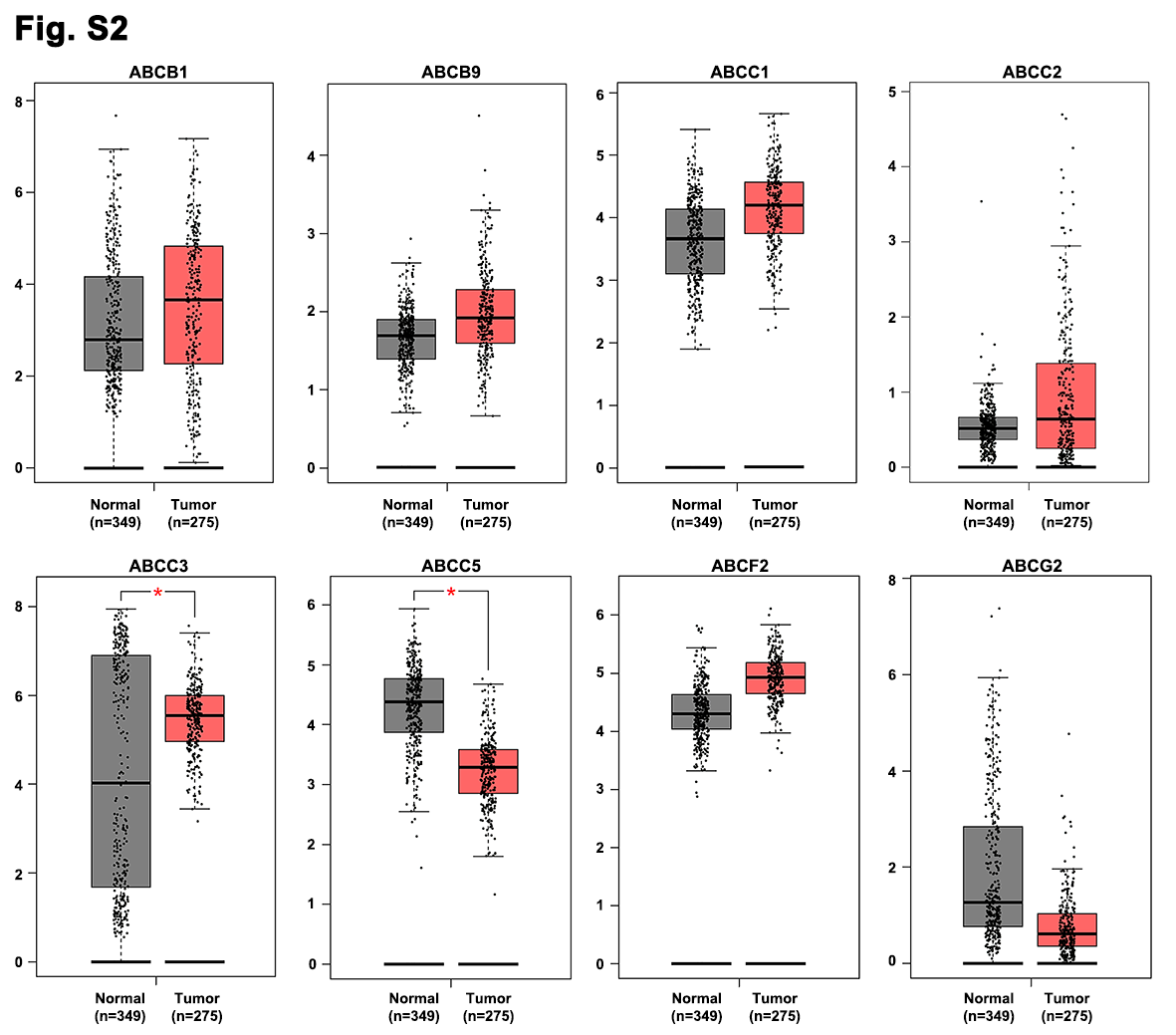

Supplement: Supplementary file 4 — Additional file 4: Fig. S2. Except for the significant higher ABCC3 mRNA and lower ABCC5 mRNA level in the CRC tissues compared with the normal mucosa, none of the mRNA expressions of ABCB1, ABCB9, ABCC1, ABCC2, ABCF2 and ABCG2 is significantly different between CRC tissues and normal mucosa based on the TCGA data analysis. * P. [file 12885_2022_10415_MOESM4_ESM.tif]

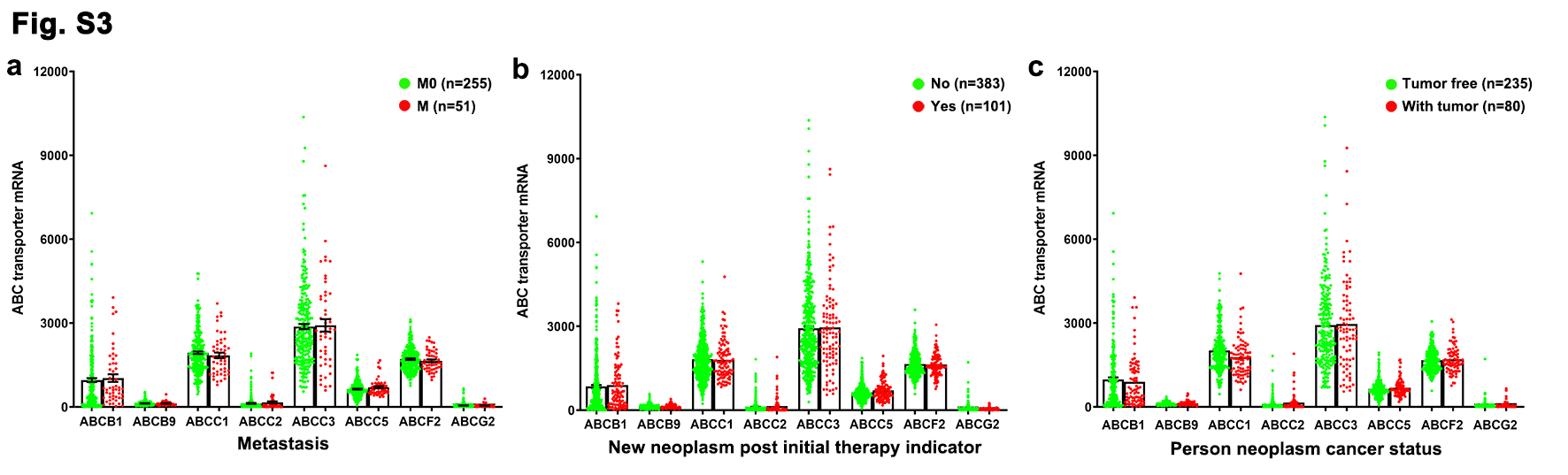

Supplement: Supplementary file 5 — Additional file 5: Fig. S3. (a) None of the mRNA expressions of ABCB1, ABCB9, ABCC1, ABCC2, ABCC3, ABCC5, ABCF2 and ABCG2 is significantly different between the metastasized and non-metastasized CRC patients. M0: no metastasis, M: metastasis. (b) None of the mRNA expressions of ABCB1, ABCB9, ABCC1, ABCC2, ABCC3, ABCC5, ABCF2 and ABCG2 is significantly different between the recurrent and non recurrent CRC patients post initial therapy. (c) None of the mRNA expressions of ABCB1, ABCB9, ABCC1, ABCC2, ABCC3, ABCC5, ABCF2 and ABCG2 in the CRC patients with tumor is significantly higher than those without tumor. [file 12885_2022_10415_MOESM5_ESM.tif]

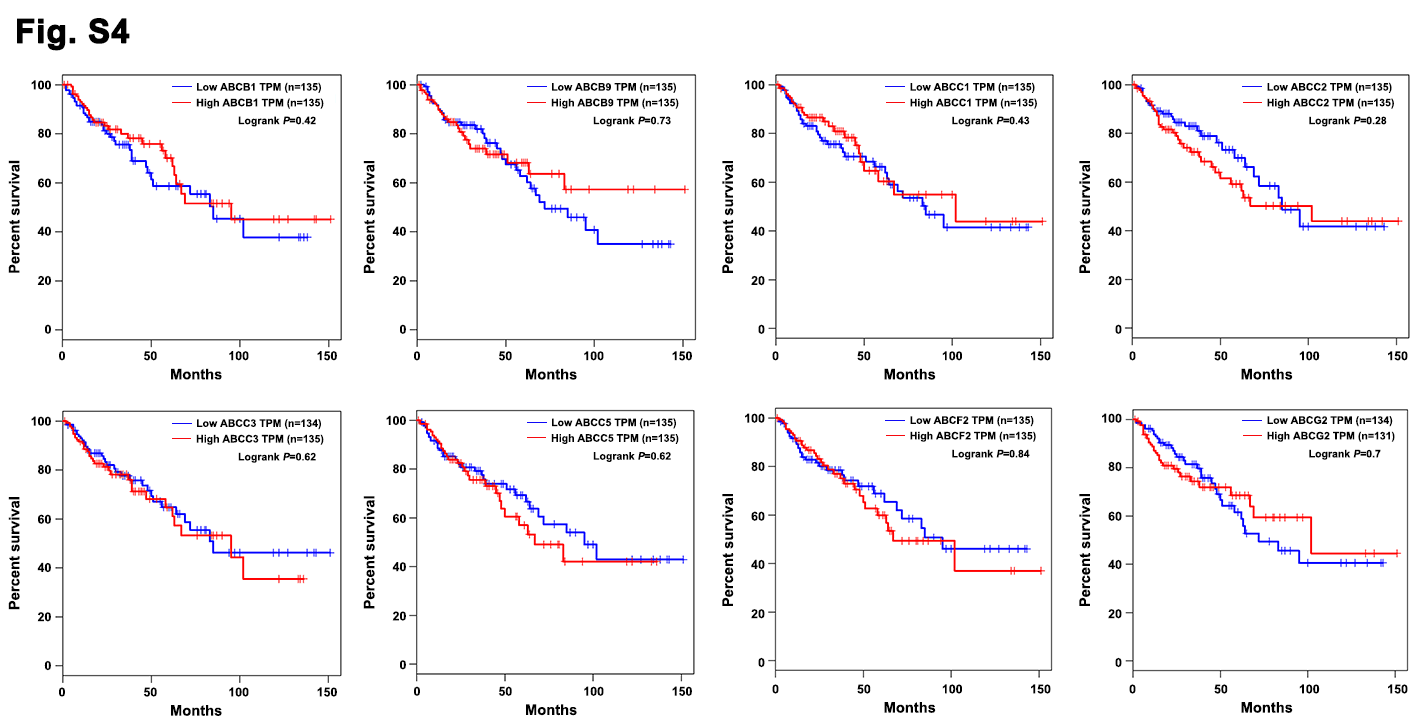

Supplement: Supplementary file 6 — Additional file 6: Fig. S4. The percent survival of the CRC patients with higherABCB1, ABCB9, ABCC1, ABCC2, ABCC3, ABCC5, ABCF2 or ABCG2 mRNA is basically the same as those with lower mRNA expression. [file 12885_2022_10415_MOESM6_ESM.tif]

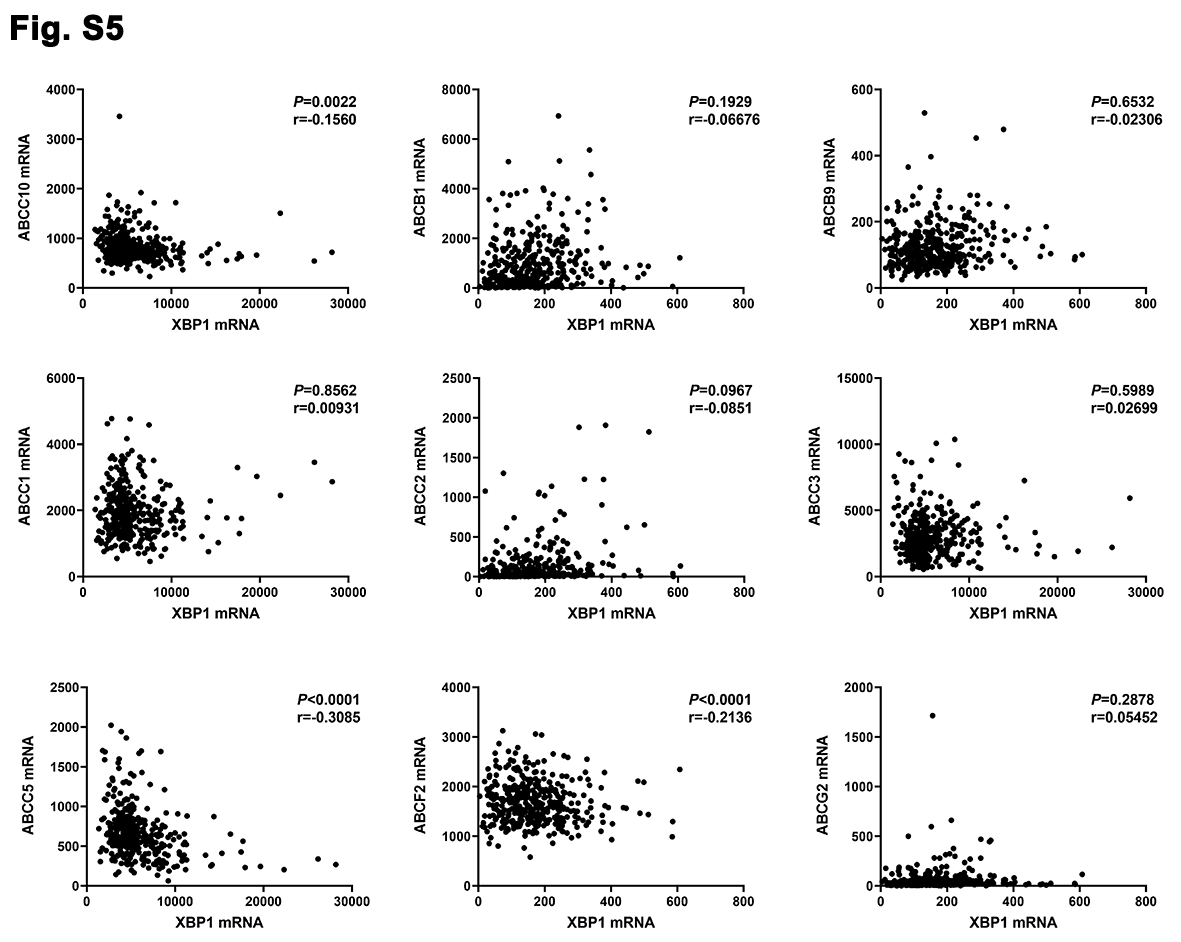

Supplement: Supplementary file 7 — Additional file 7: Fig. S5. Analysis on the correlation between ABC transporter mRNA level and XBP-1, a mediator within IRE1α pathway, was made based on TCGA data. ABCC10, ABCC5 and ABCF2 negatively correlated with XBP-1. [file 12885_2022_10415_MOESM7_ESM.tif]

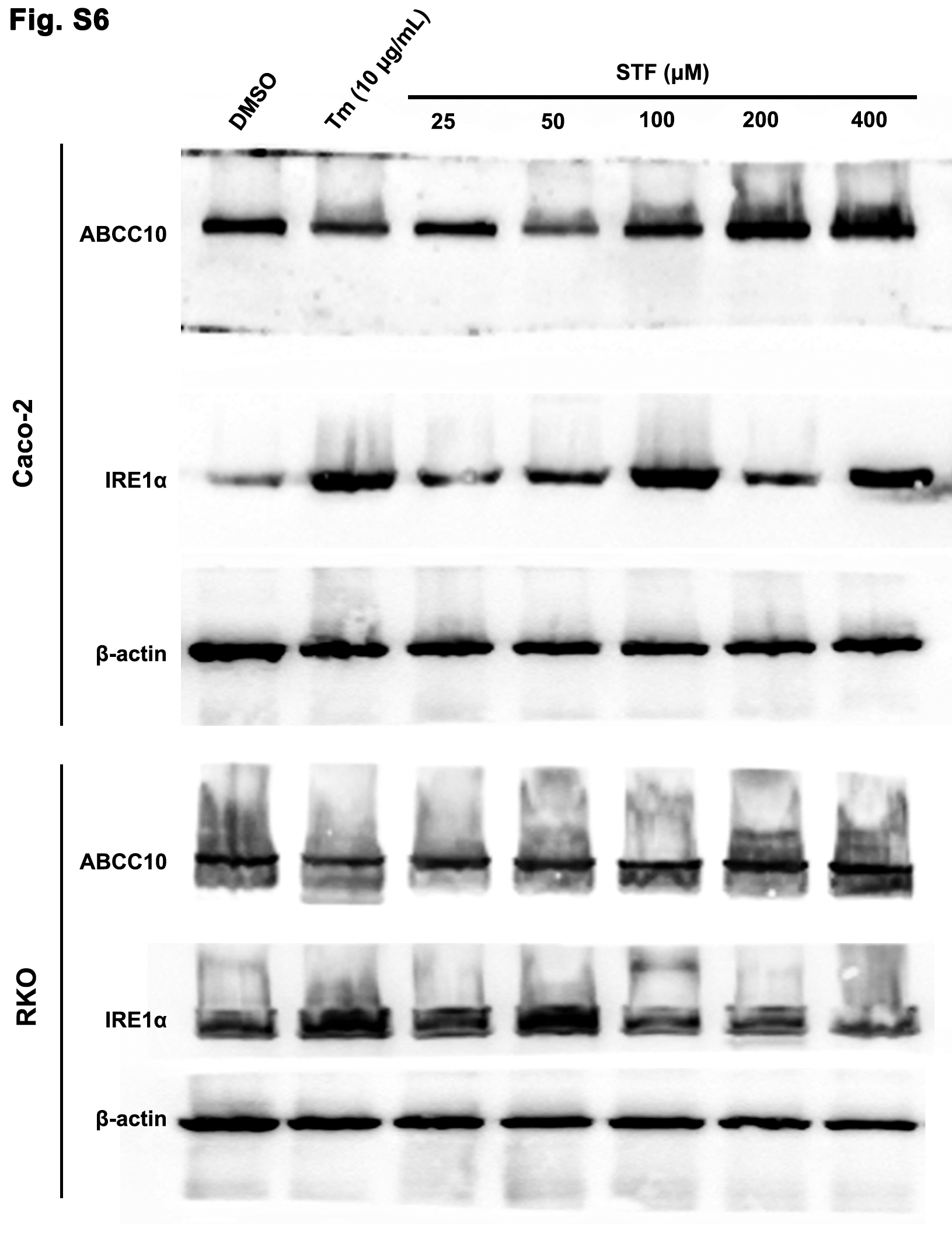

Supplement: Supplementary file 8 — Additional file 8: Fig. S6. Caco-2 and RKO cells were treated with Tm (10 μg/mL) or STF (25, 50, 100, 200 and 400 μM). IRE1α pathway is inactivated upon the treatment of 200 or 400 μM of STF, by which ABCC10 is elevated. Therefore, 200 μM of STF was used in the further experiments. [file 12885_2022_10415_MOESM8_ESM.tif]

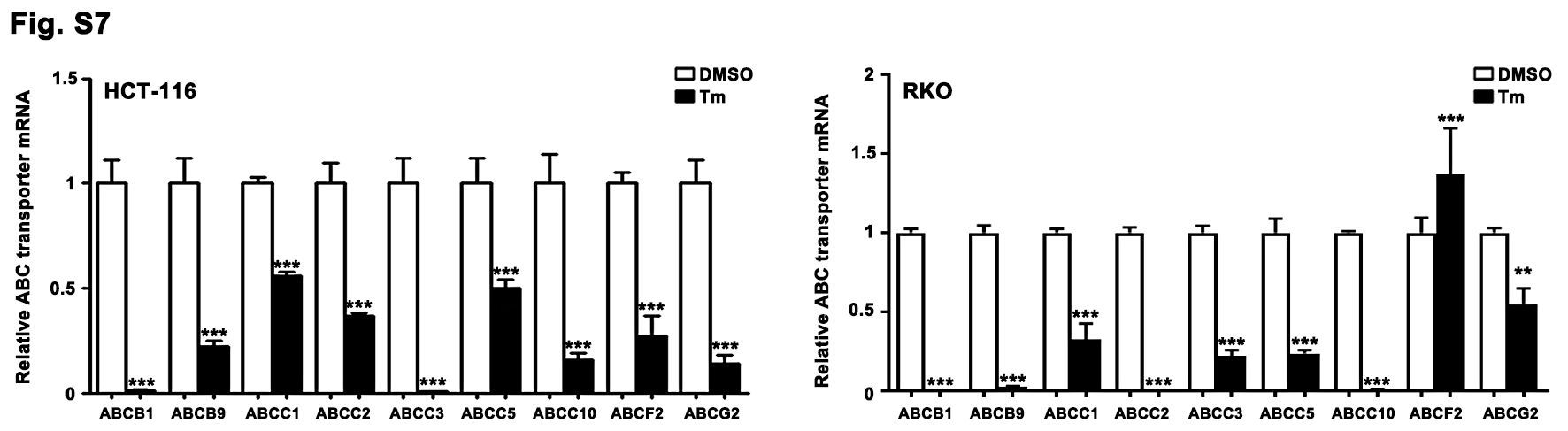

Supplement: Supplementary file 9 — Additional file 9: Fig. S7. Intense ERS/UPR significantly down-regulates mRNA expression of multiple ABC transporters in RKO and HCT-116 cells. RKO and HCT-116 cells are exposed to high dose of Tm (10 µg/mL). ABC transporters including ABCB1, ABCB9, ABCC1, ABCC2, ABCC3, ABCC5, ABCC10, ABCF2 and ABCG2 are remarkably downregulated in Tm-treated HCT-116 cells compared with controls. ABCB1, ABCB9, ABCC1, ABCC2, ABCC3, ABCC5, ABCC10 and ABCG2 were down-regulated in Tm-treated RKO cells while ABCF2 were up-regulated compared with controls. n=3. * P<0.001. [file 12885_2022_10415_MOESM9_ESM.tif]

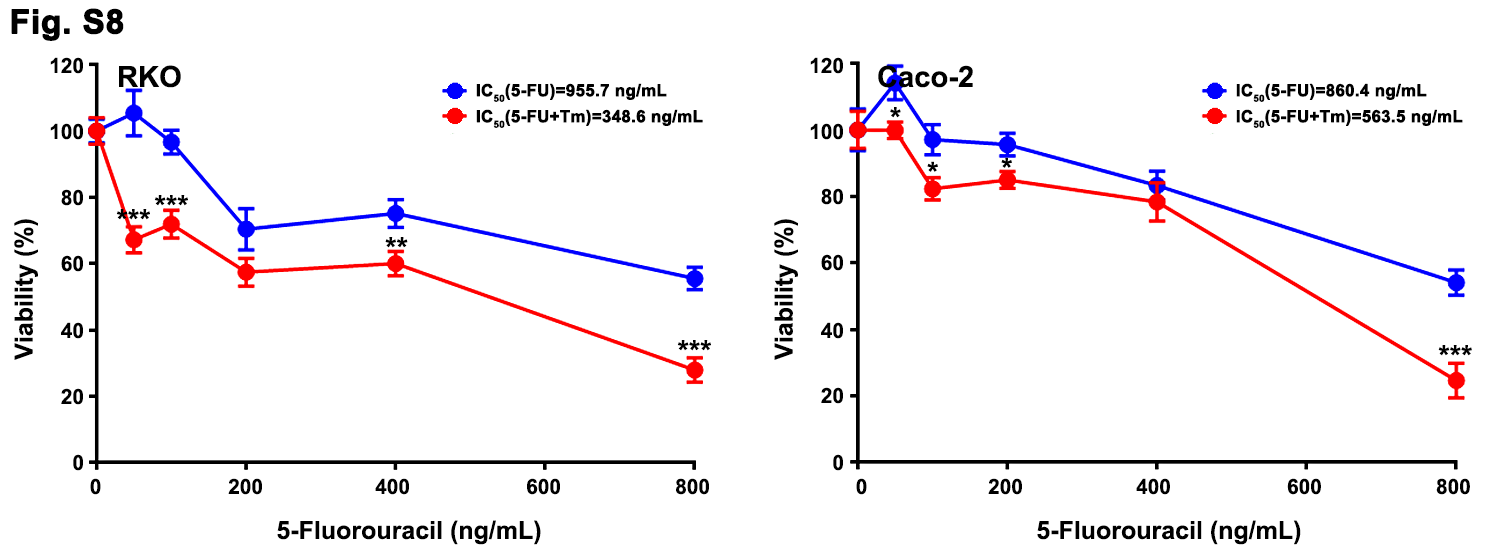

Supplement: Supplementary file 10 — Additional file 10: Fig. S8. RKO and Caco-2 cells were exposed to a gradient of 5-Fluorouracil (5-FU) in the absence or presence of Tm (10 µg/mL) for 24 h. 5-FU IC50 is significantly decreased in response to Tm. n=6. * P [file 12885_2022_10415_MOESM10_ESM.tif]

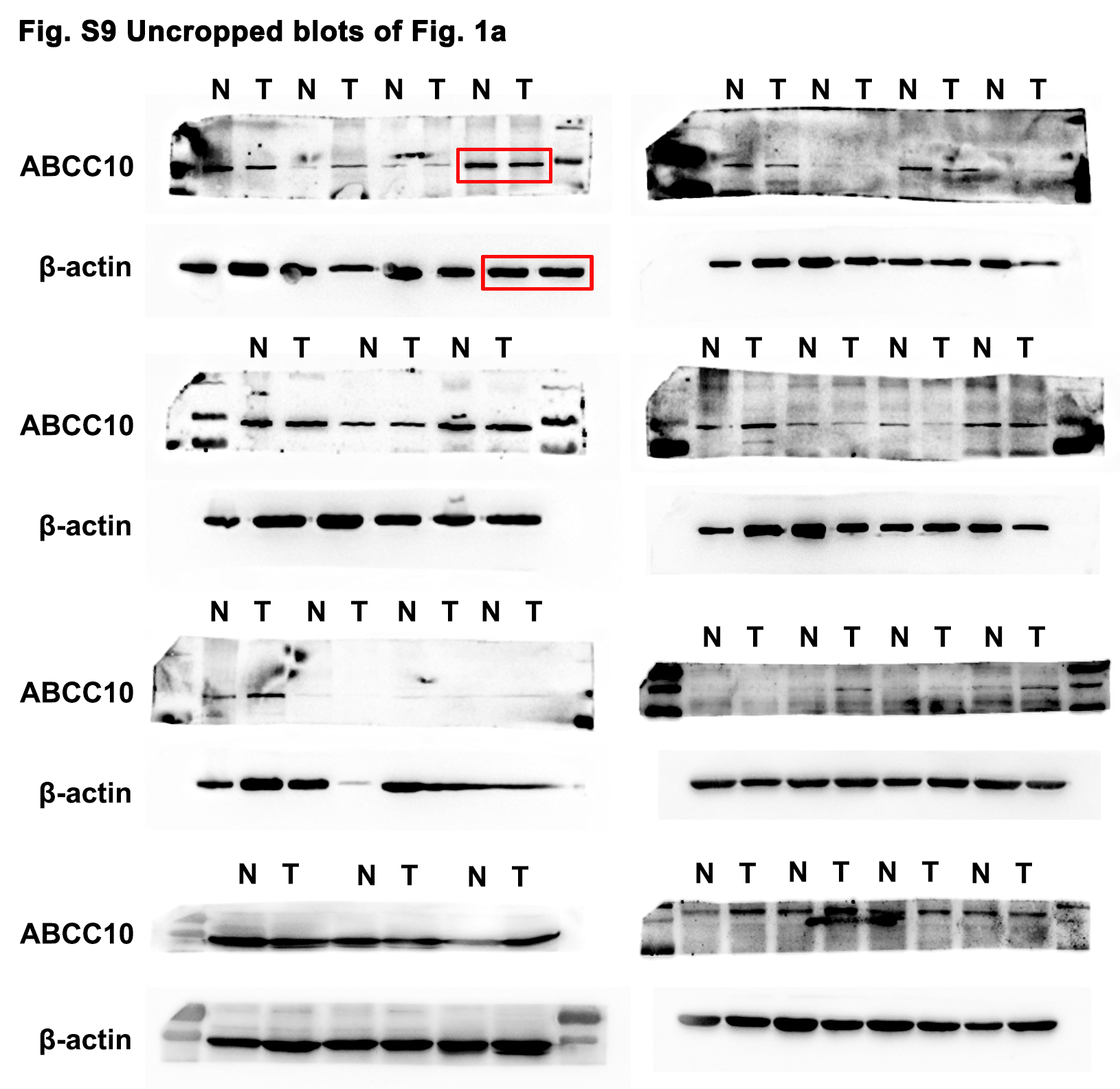

Supplement: Supplementary file 11 — Additional file 11: Fig. S9. Original data: Uncropped western blot images of Fig. 1a. [file 12885_2022_10415_MOESM11_ESM.tif]

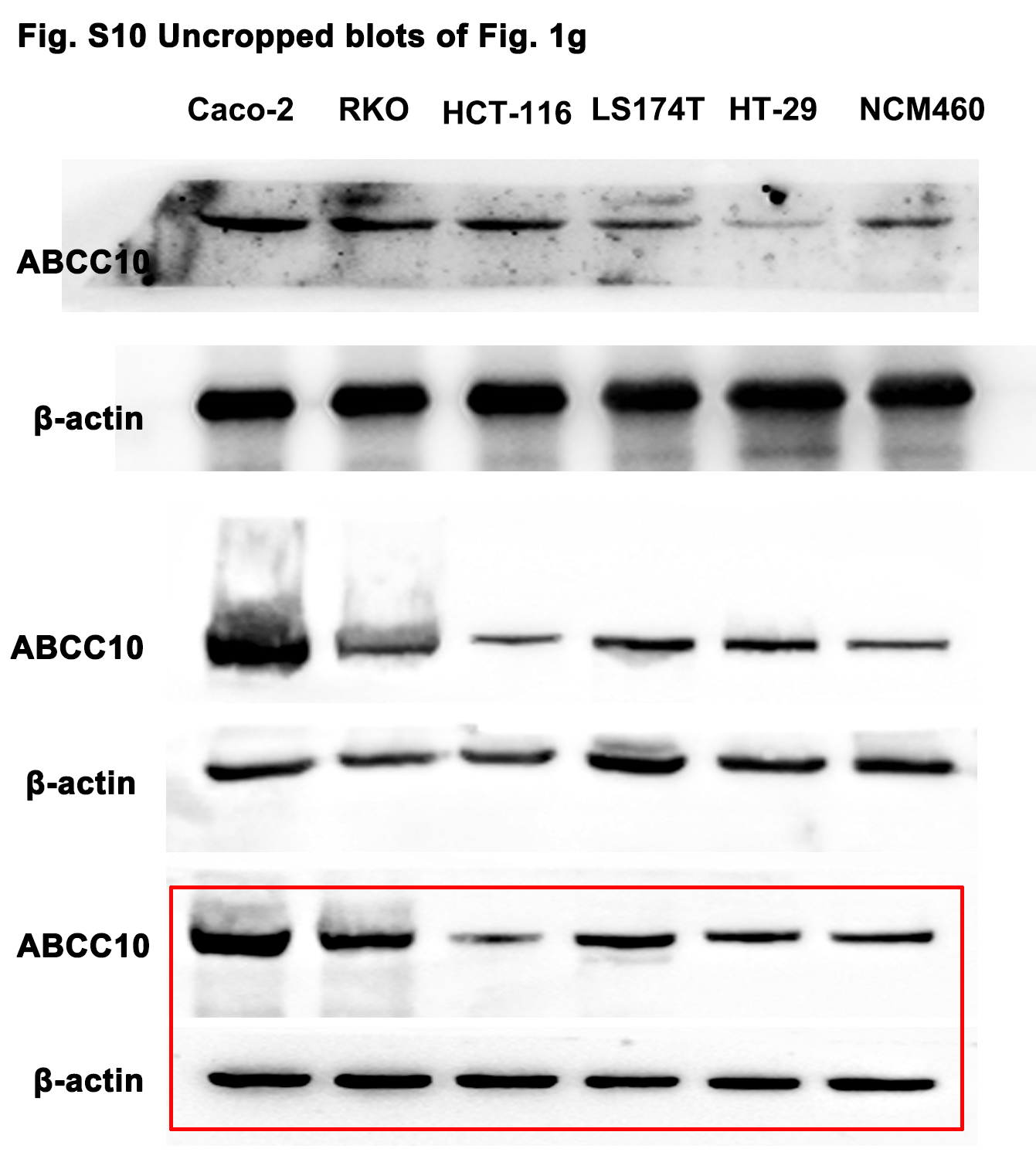

Supplement: Supplementary file 12 — Additional file 12: Fig. S10. Original data: Uncropped western blot images of Fig. 1g. [file 12885_2022_10415_MOESM12_ESM.tif]

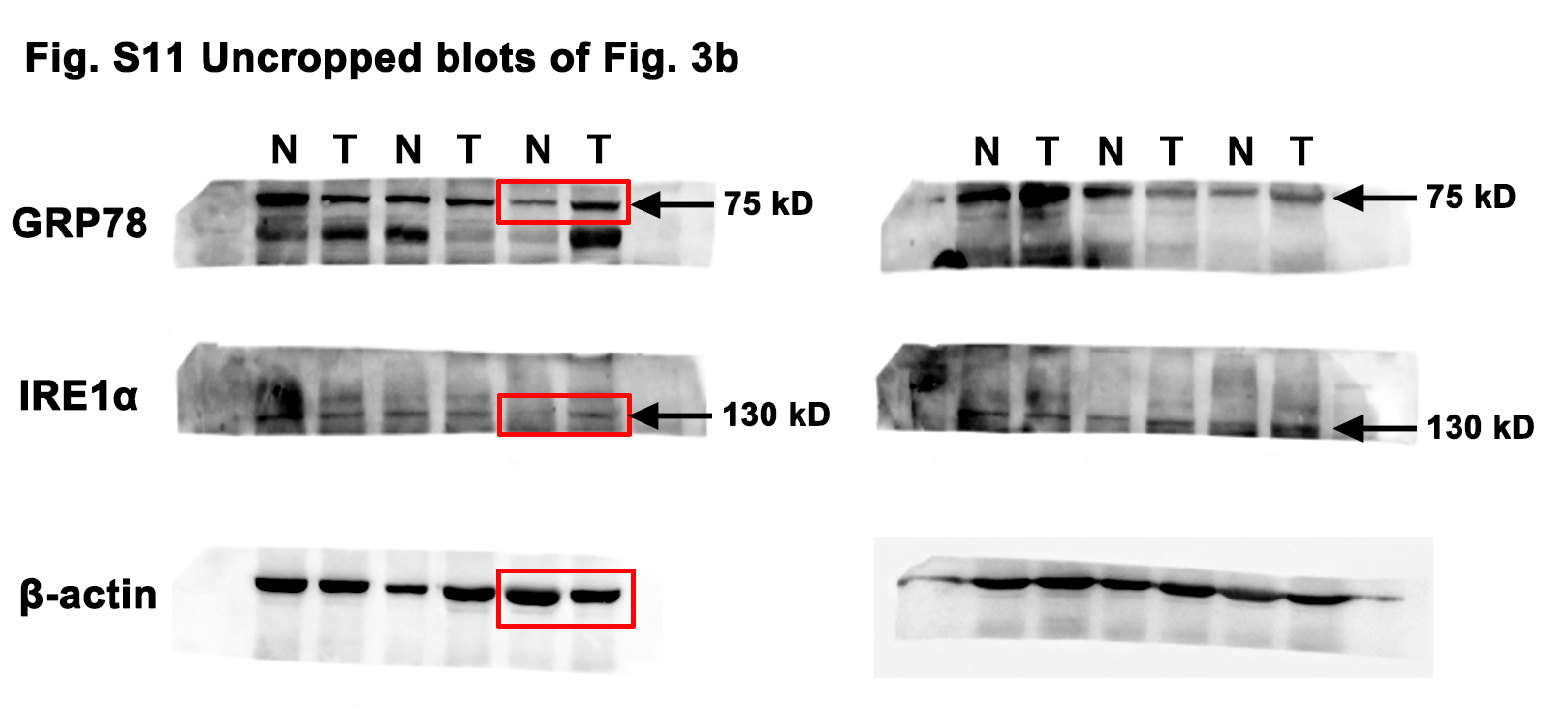

Supplement: Supplementary file 13 — Additional file 13: Fig. S11. Original data: Uncropped western blot images of Fig. 3b. [file 12885_2022_10415_MOESM13_ESM.tif]

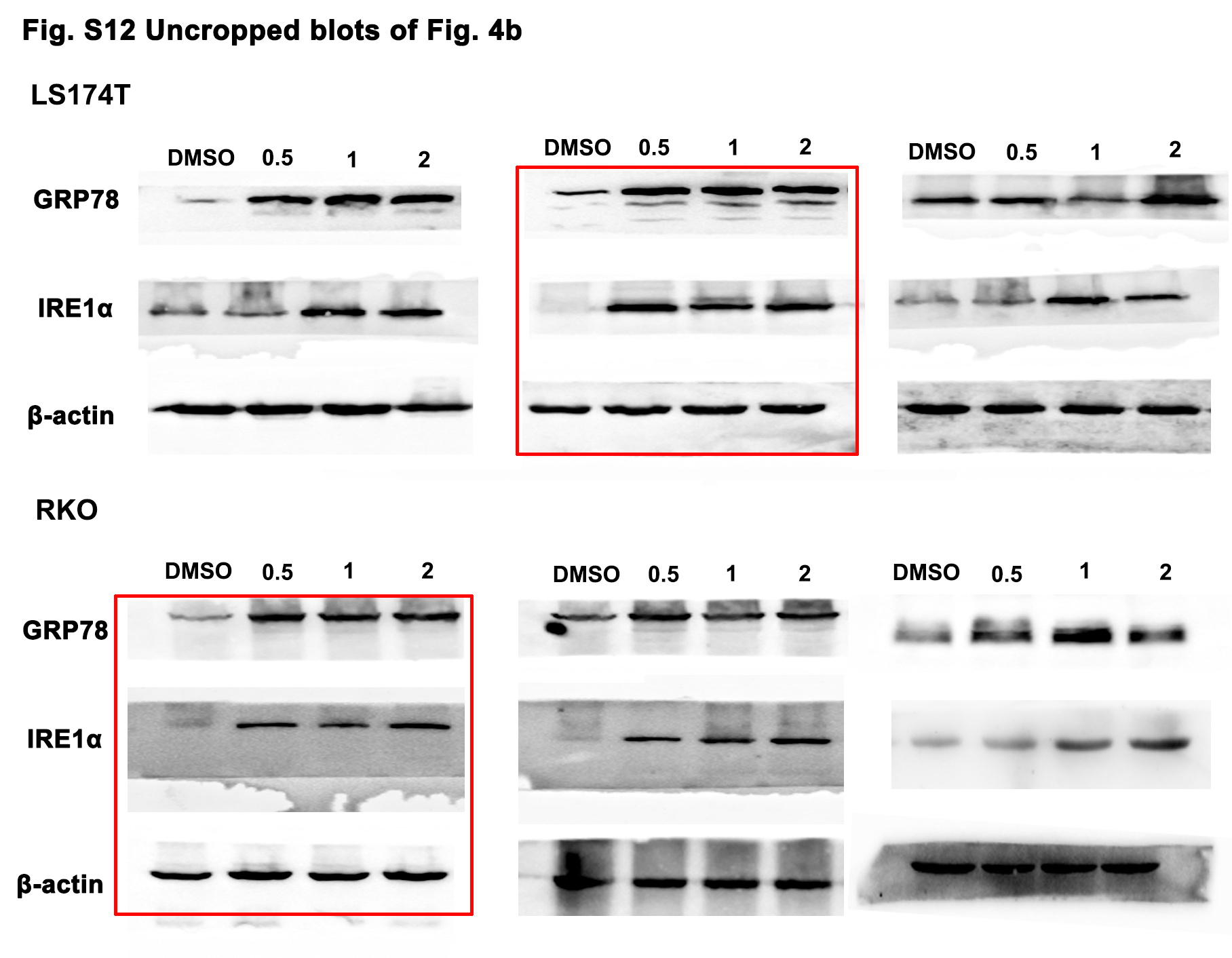

Supplement: Supplementary file 14 — Additional file 14: Fig. S12. Original data: Uncropped western blot images of Fig. 4b [file 12885_2022_10415_MOESM14_ESM.tif]

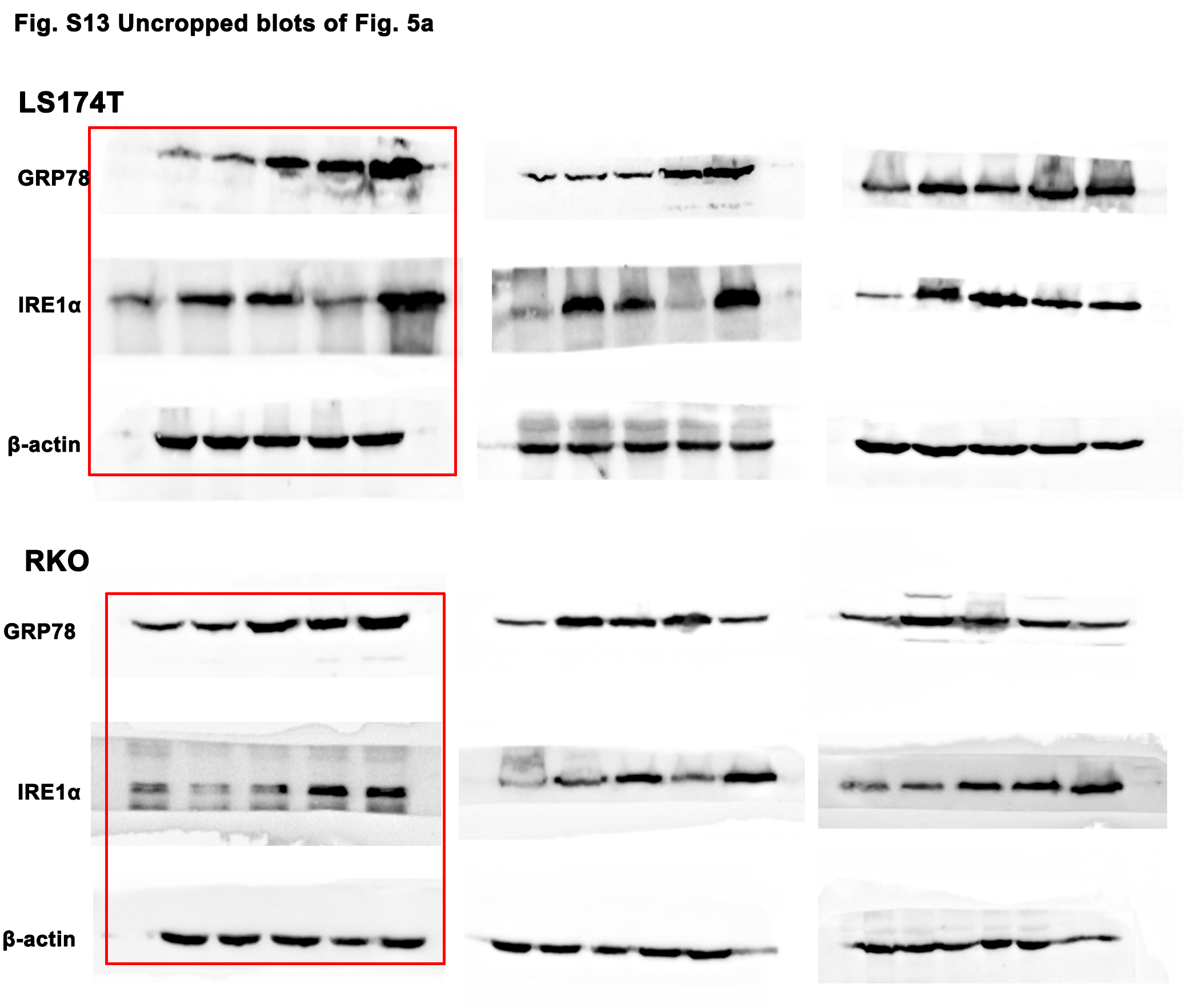

Supplement: Supplementary file 15 — Additional file 15: Fig. S13. Original data: Uncropped western blot images of Fig. 5a. [file 12885_2022_10415_MOESM15_ESM.tif]

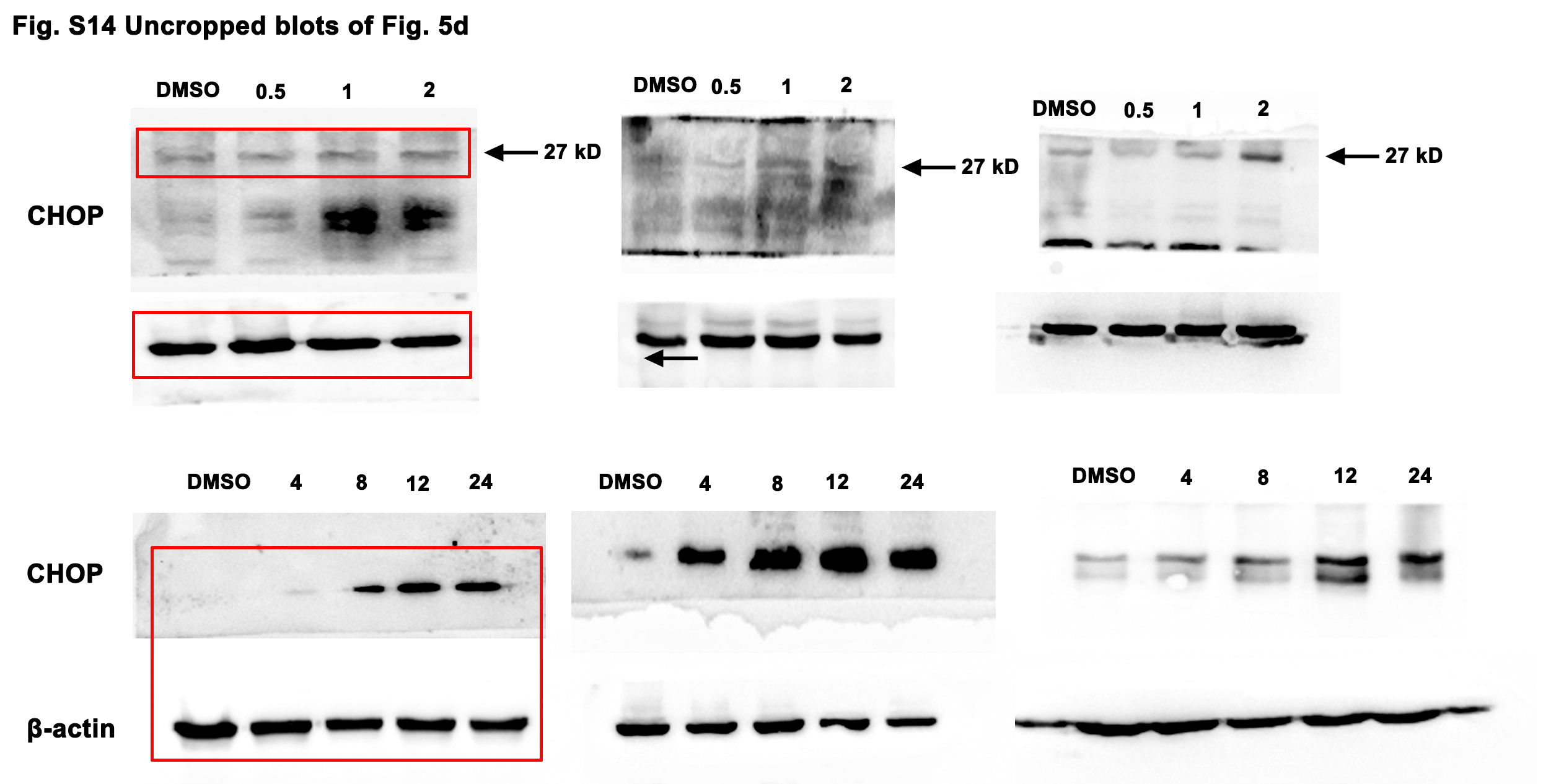

Supplement: Supplementary file 16 — Additional file 16: Fig. S14. Original data: Uncropped western blot images of Fig. 5d. [file 12885_2022_10415_MOESM16_ESM.tif]

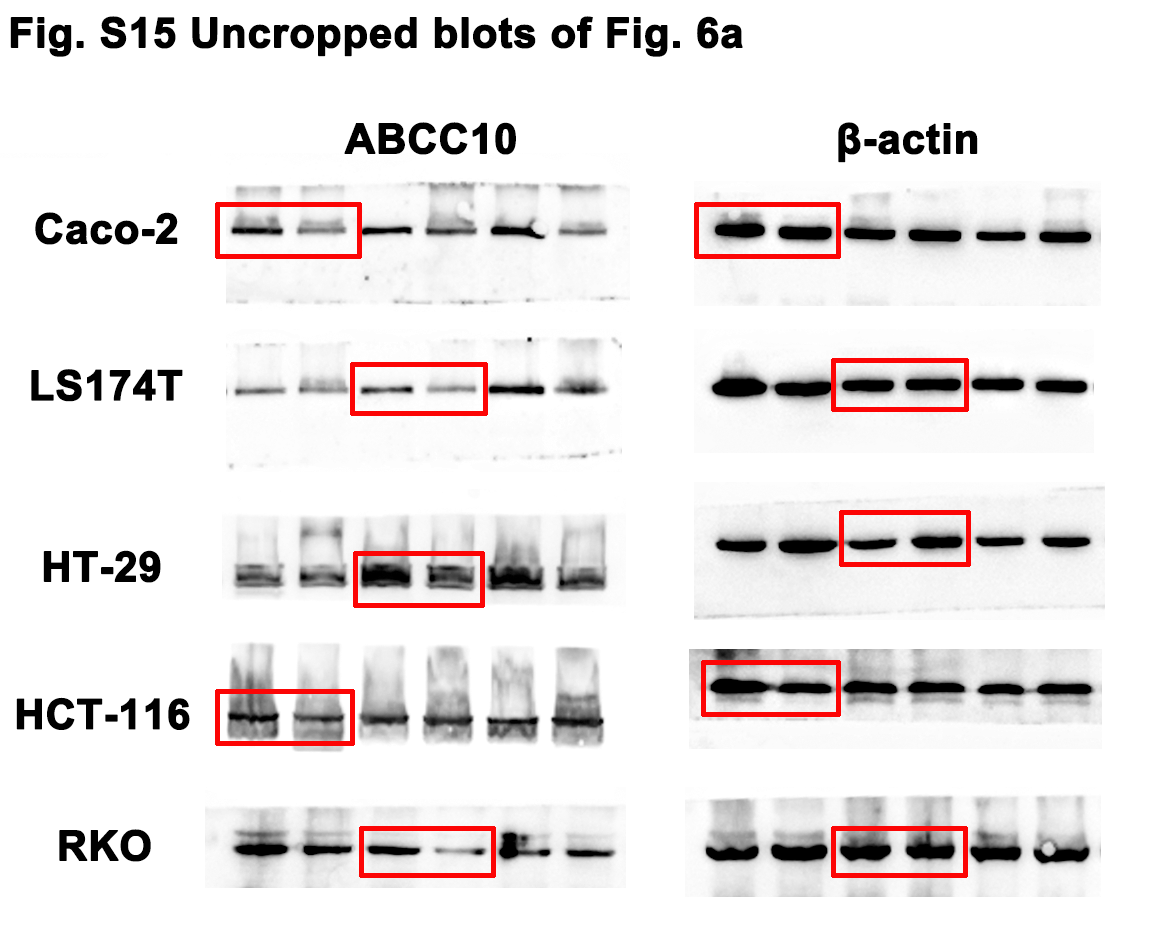

Supplement: Supplementary file 17 — Additional file 17: Fig. S15. Original data: Uncropped western blot images of Fig. 6a. [file 12885_2022_10415_MOESM17_ESM.tif]

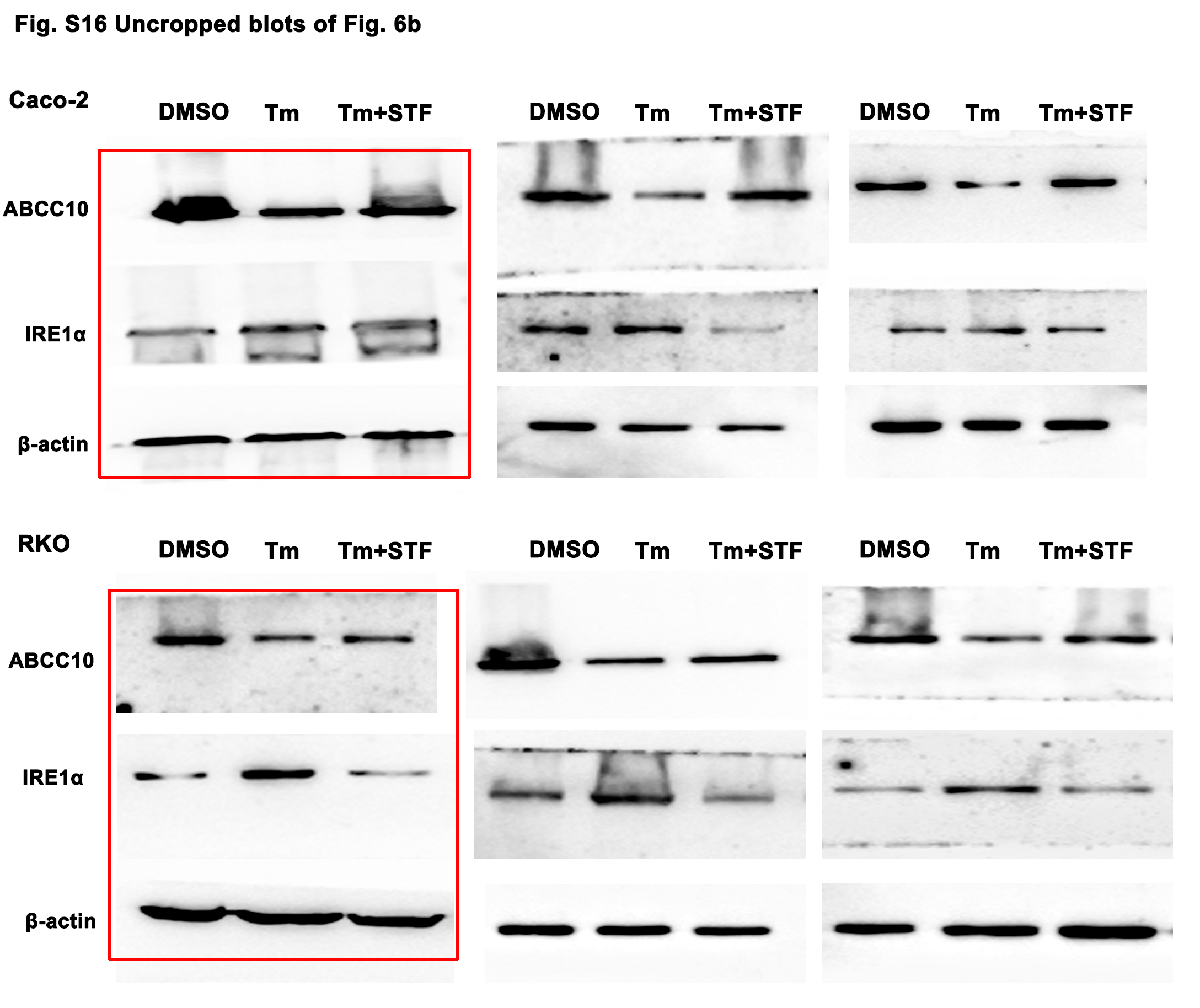

Supplement: Supplementary file 18 — Additional file 18: Fig. S16. Original data: Uncropped western blot images of Fig. 6b. The framed regions of the blots in Fig. S9~S16 are used in the manuscript. Since the blots were cut prior to hybridisation with antibodies, the provided uncropped images are not of full length. [file 12885_2022_10415_MOESM18_ESM.tif]
